# Preventing Salt Precipitation in CO_2_ Storage Processes in Saline Aquifers: Dissolved-Water CO_2_ Injection Method

**DOI:** 10.1021/acs.energyfuels.4c05249

**Published:** 2025-02-18

**Authors:** Ali Papi, Amir Jahanbakhsh, M. Mercedes Maroto-Valer

**Affiliations:** † Research Centre for Carbon Solutions (RCCS), School of Engineering and Physical Sciences, Heriot-Watt University, Edinburgh EH14 4AS, U.K.; ‡ Industrial Decarbonisation Research and Innovation Centre (IDRIC), Heriot-Watt University, Edinburgh EH14 4AS, U.K.

## Abstract

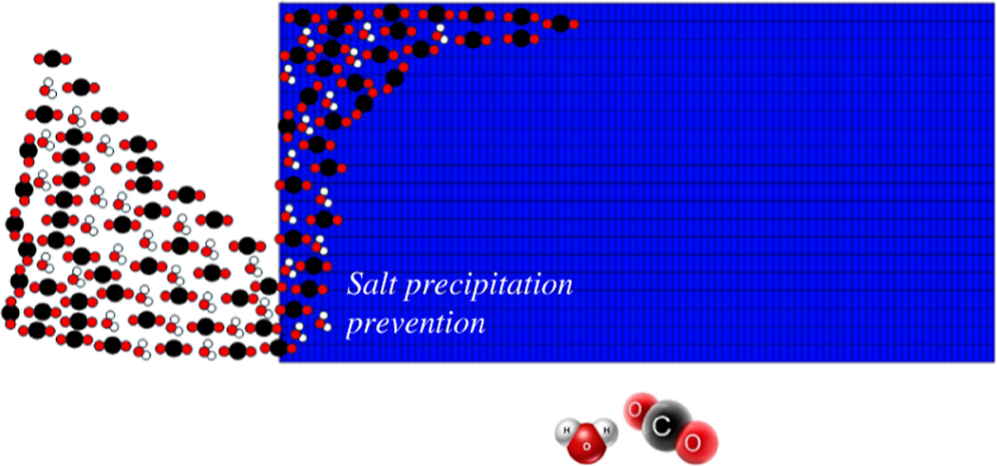

CO_2_ storage in geological formations, particularly deep saline aquifers, is a critical component of carbon capture and storage technology, offering significant potential for mitigating greenhouse gas emissions. However, high salinity of these aquifers poses the risk of salt precipitation, leading to pressurization and injectivity reduction. Developing a method to prevent salt precipitation remains a challenge, and this is an area that this study is focused on. Dissolved-water CO_2_ injection (dwCO_2_ injection) is proposed here as a novel method to prevent salt precipitation where water is dissolved in CO_2_ before injection into an aquifer. Presence of water in the CO_2_ stream prevents more dissolution of water into CO_2_ (evaporation) and, hence, prevents salt precipitation. Before presenting this method and in order to provide a good mechanistic understanding of the interactions involved in a CO_2_ storage process, six different scenarios are examined using the CMG-GEM simulator within a carbonate aquifer. The results showed that saturating CO_2_ with water reduced the precipitation nearly to zero, and dissolving 2000 ppmv water decreased the salt precipitation to one-third. It should be noted that injection of humid CO_2_ requires special methods to tackle the potential challenges, including corrosion and hydrate formation risks, and the paper also discusses them.

## Introduction

CO_2_ geological storage is a promising technology for mitigating greenhouse gas emissions.^1,2^ This can include storing CO_2_ in partially depleted or depleted oil and gas reservoirs, coal formations, or shallow and deep saline aquifers.^3−6^ Deep saline aquifers with high availability are known to have the greatest storage potential for CO_2_ storage, at least estimated to be at 1000 GtCO_2_.^7−9^ As CO_2_ is injected into the subsurface, it is involved into a number of physical and chemical processes with the resident fluids and porous rock.^8,10−13^ These include structural, residual, solubility, and mineralization trapping.^14−17^ Additionally, the high salinity of saline aquifers presents the threat of salt precipitation to CO_2_ storage processes in these environments, which is caused by the evaporation of water near the wellbore region.^18−20^ Furthermore, evaporation causes a capillary pressure gradient in the porous medium that induces a brine capillary backflow in the system and intensifies salt precipitation.^21−23^ Salt precipitation can plug the pores near the well, reduce the well injectivity, and cause pressurization.^24−26^ More information regarding these geological interactions is presented in the Supporting Information (Theoretical Background).

Water-saturated CO_2_ injection (wsCO_2_ injection) has been recently proposed in the context of carbon capture, storage, and utilization (CCUS) as an enhanced oil recovery method to increase oil production and improve CO_2_ storage.^27−30^ In this method, CO_2_ is saturated with water at the reservoir pressure and temperature conditions before injection into the reservoir. When CO_2_ is saturated with water, in the case of miscibility (either first-contact or multiple-contact miscibility^31^), CO_2_ is condensed into the oil phase, and due to this condensation, water is dropped out of the CO_2_ dense phase. As the dropped-out water occupies a portion of the reservoir pore space, fewer pathways remain open for the CO_2_ dense phase compared to when a dry CO_2_ injection case is considered. Hence, relative permeability and mobility of the CO_2_ dense phase are reduced, leading to a better sweep efficiency, oil recovery, and CO_2_ storage. These findings were confirmed in coreflood experiments of oil-saturated samples, comparing wsCO_2_ injection to dry CO_2_ injection at near-miscibility conditions.^27^ In another study, it was also concluded that miscible conditions and low injection rates improved the efficiency of the method in enhancing oil recovery and CO_2_ storage.^28^ Similar results were achieved in further work at tertiary oil recovery conditions.^30^ Having said these, none of these studies considered the effects on salt precipitation. Additionally, they did not discuss the feasibility of their wsCO_2_ injection method and how it should be implemented on the field scale to be practicable, considering the flow assurance implications that would arise from this method.

There is no universally accepted water content in a CO_2_ injection project, and it depends on the process.^32,33^ The industry generally favors an inherently safe design when it comes to process safety. This approach prioritizes the active elimination of hazards over passively managing them.^34^ In this context, reducing the water content in CO_2_ is preferred to mitigate the risks of corrosion, hydrate formation, and two-phase flow conditions.^32^ Unless there is a specific technical or economic advantage in injecting humid CO_2_, the current recommendations in place are to maintain the CO_2_ water content as dry as possible, below 50 molar ppm.^33,35^ This minimizes the need to address the possible risks and consequences. However, various water contents from 50 to 650 ppm have been reported in different CO_2_ transportation projects across the world.^33,36−41^ The Sleipner CO_2_ storage project has been operated under water-saturated conditions with the benefit of corrosion resistive alloys (CRAs).^32,33,36,42^ Corrosion has been identified as one of the biggest challenges arising from the humid injection of CO_2_.

**Figure 1:**
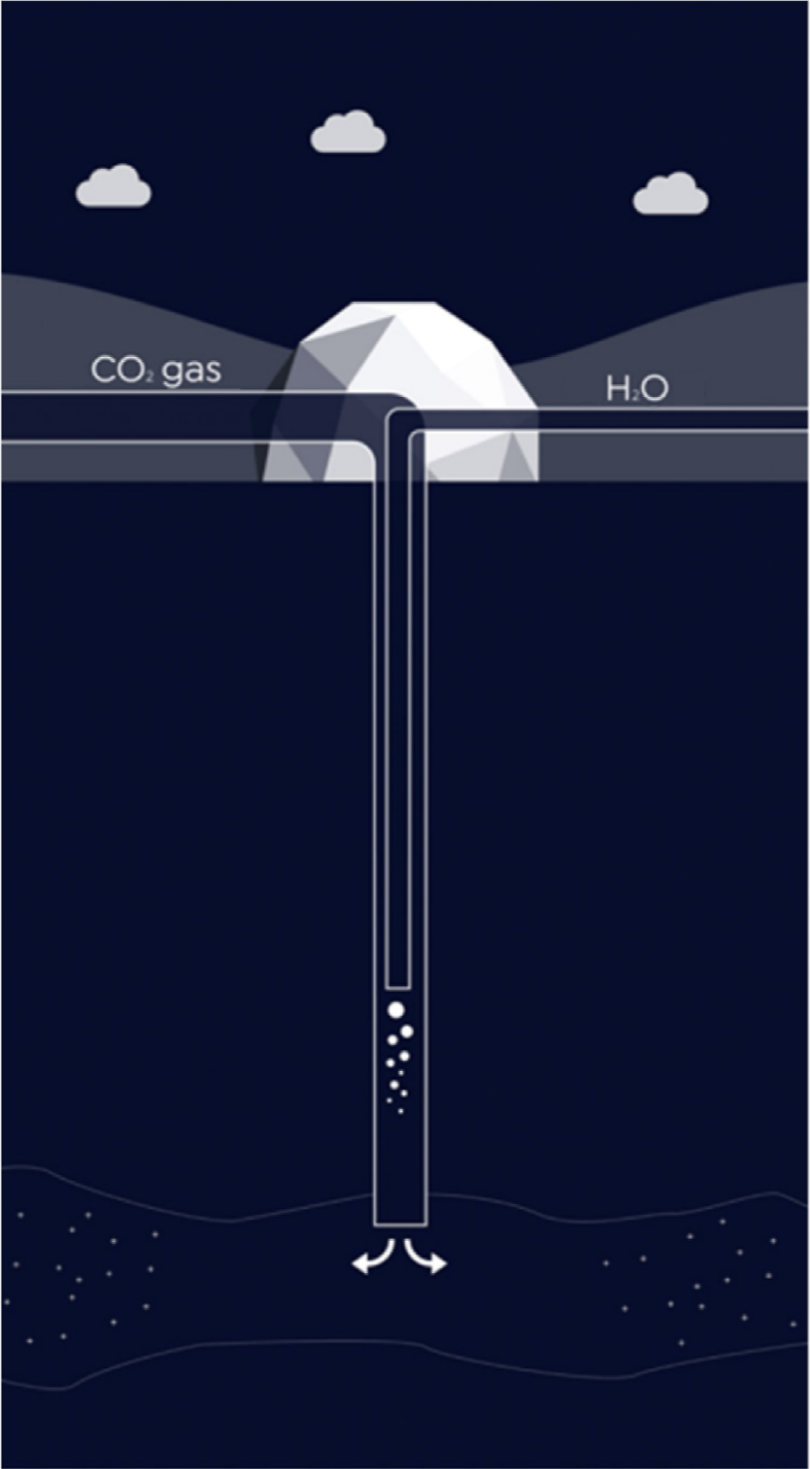
Injecting CO_2_ at humid conditions by dissolving water into CO_2_ stream within the well using a disperser. Reproduced or adapted with permission from ref (44). Copyright 2024 Elsevier.

There are two ways to conduct CO_2_ injection under humid conditions. In the first method, water can be injected into the CO_2_ stream at the wellhead in the last stage of transportation.^43^ Water may be dissolved in CO_2_ by spraying it deep into the well using a specific disperser (Figure 1).^44,45^ This method was practiced in CarbFix CO_2_ injection project where CO_2_ was dissolved into water in the form of small bubbles inside the well before entering the reservoir.^46,47^ A sparger was used to disperse CO_2_ in water.^48,49^ By adopting this method, challenges such as corrosion are prohibited, and recommendations on minimum water content in the CO_2_ stream during transportation can be appropriately regarded.

**Figure 2:**
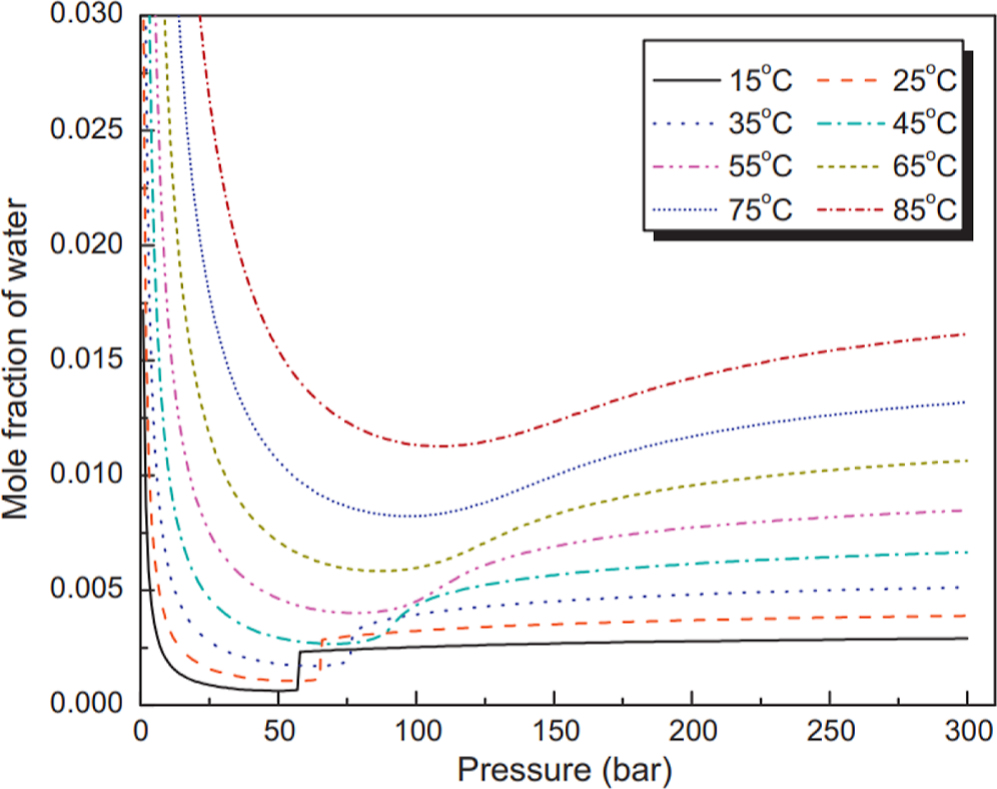
Water solubility in CO_2_ as a function of pressure and temperature. Various water contents can be tolerated in a CO_2_ stream without water dropout and the risk of corrosion. Reproduced or adapted with permission from ref (50). Copyright 2011 Elsevier.

The second method is transporting CO_2_ under humid conditions. This method is based on the fact that corrosion can become an issue only when the water is present in the CO_2_ stream in the form of free water.^32^ That is when different corrosive acids may be formed in the aqueous phase.^33^ The plot of water solubility in CO_2_ at different temperatures and pressures is shown in Figure 2.^50^ Depending on the thermodynamic conditions of the injection process, different amounts of water content in the CO_2_ phase can be tolerated as dissolved, without free water dropout and corrosion concerns.^51,52^ With this second method, the project costs can be reduced, as a dehydration stage may not be required. This option was practiced in the Gorgon carbon capture and storage (CCS) project.^53^ However, the project stopped due to the lack of an appropriate engineering design. A summary of this project is given toward the end of this study. Performing injection in this second method is highly operational-dependent and requires accurate process engineering and design, such as defining the maximum allowable water content in the CO_2_ phase without free water dropout. More information about the challenges and feasibility of this method is discussed in Supporting Information (Theoretical Background).

Additionally, in the case of free water formation, the use of CRAs has shown to work well in the prevention of corrosion in all the noncomplex mixtures of CO_2_, i.e., the mixtures that exclude NO_*x*_ and SO_*x*_.^54^ Moreover, corrosion inhibitors are an emerging technology under study in the field of CCS, with ongoing efforts to develop solutions tailored to its specific requirements.^55,56^

This study highlights a technical advantage of the humidity presence in the CO_2_ stream to eliminate or mitigate salt precipitation. A number of methods have been suggested to mitigate salt precipitation in CO_2_ storage processes.^57−59^ However, salt precipitation still remains a challenge, both technically and economically.^60^ Acid washing, fresh water injection, or injecting a low-salinity water slug before and after CO_2_ injection have been suggested to reduce salt precipitation.^21,61−63^ Injecting a low-salinity water slug before CO_2_ injection creates a barrier between the injected CO_2_ and the resident brine and reduces salt precipitation. On the other hand, injecting the low-salinity water slug after the CO_2_ injection aids in dissolving and washing out any precipitated salt.^63^ Having said that, these methods that are temporary solutions can cause long-term issues such as clay swelling.^64^ Some researchers have suggested making the near wellbore region nonwet to water can reduce the possibility of salt accumulation in that area.^65,66^ This is because the capillary backflow phenomenon that brings a massive amount of brine back to the injection point happens through the connected liquid films attached to the water-wet rock surfaces.^21^ However, if the rock is nonwet to water, water will not attach to the rock surface, and this film flow will be disconnected. Therefore, ex situ salt precipitation cannot happen, leading to a reduction in salt precipitation.^65,66^

This work proposes dissolved-water CO_2_ injection (dwCO_2_ injection) as a remedy to solve the issue of salt formation near the wellbore during injection of CO_2_ in deep saline aquifers and discusses the related challenges. To the best of our knowledge, it is the first time that dwCO_2_ injection is proposed as a solution to salt precipitation, and this method has not been explored in the literature for this specific application. In this method, less than 1% water content is dissolved into the CO_2_ stream before injection into the reservoir. The reason why a different name than water-saturated CO_2_ injection (wsCO_2_ injection) has been chosen is that the proposed dissolved-water CO_2_ injection (dwCO_2_ injection) is a broader naming convention. In the second injection method, the CO_2_ stream cannot be saturated with water at reservoir pressure and temperature due to flow assurance challenges such as corrosion, as explained in the Supporting Information. So, practically, the CO_2_ stream would not be at its reservoir water saturation but rather at a lower dissolved water content, unless other engineering measures are in place such as the use of a CRA or an appropriate corrosion inhibitor. This study suggests that with some engineering efforts, salt precipitation can be prohibited appropriately. The industry faces a trade-off between deploying such engineering measures and having to deal with salt precipitation maintenance costs. It should be especially noted that the damage to the reservoir due to salt precipitation can be unsustainable in terms of reservoir and asset management.

In this paper, different simulation scenarios of a CO_2_ storage process are examined using CMG-GEM compositional simulator to demonstrate the mechanisms and phenomena involved in a CO_2_ storage process in the subsurface.^67^ This is to give background knowledge of the process before proposing the salt precipitation remediation strategy. Six scenarios are considered in this study; in scenario 1, water evaporation and salt precipitation are investigated. In scenario 2, CO_2_ dissolution effects are considered. In scenarios 3 and 4, the effect of geochemical reactions is added to the previous scenarios to observe all the fluid/rock interactions within a single CO_2_ storage process. The simulation results of scenarios 3 and 4 will help to investigate the mutual effects of evaporation and salt precipitation phenomena and the CO_2_ trapping mechanisms. Calcite and dolomite as the most dominant minerals of carbonate rocks that have shown higher reactivity potentials are considered the hosting environment. In scenario 5, the effect of capillary forces on the extent of salt precipitation is investigated. After deciphering and demonstrating the principles behind all the mechanisms, dwCO_2_ injection as a solution to prevent salt precipitation is proposed through scenario 6, and its effect on salt precipitation reduction is demonstrated.

## Materials and Methods

### Model Construction

**Figure 3:**
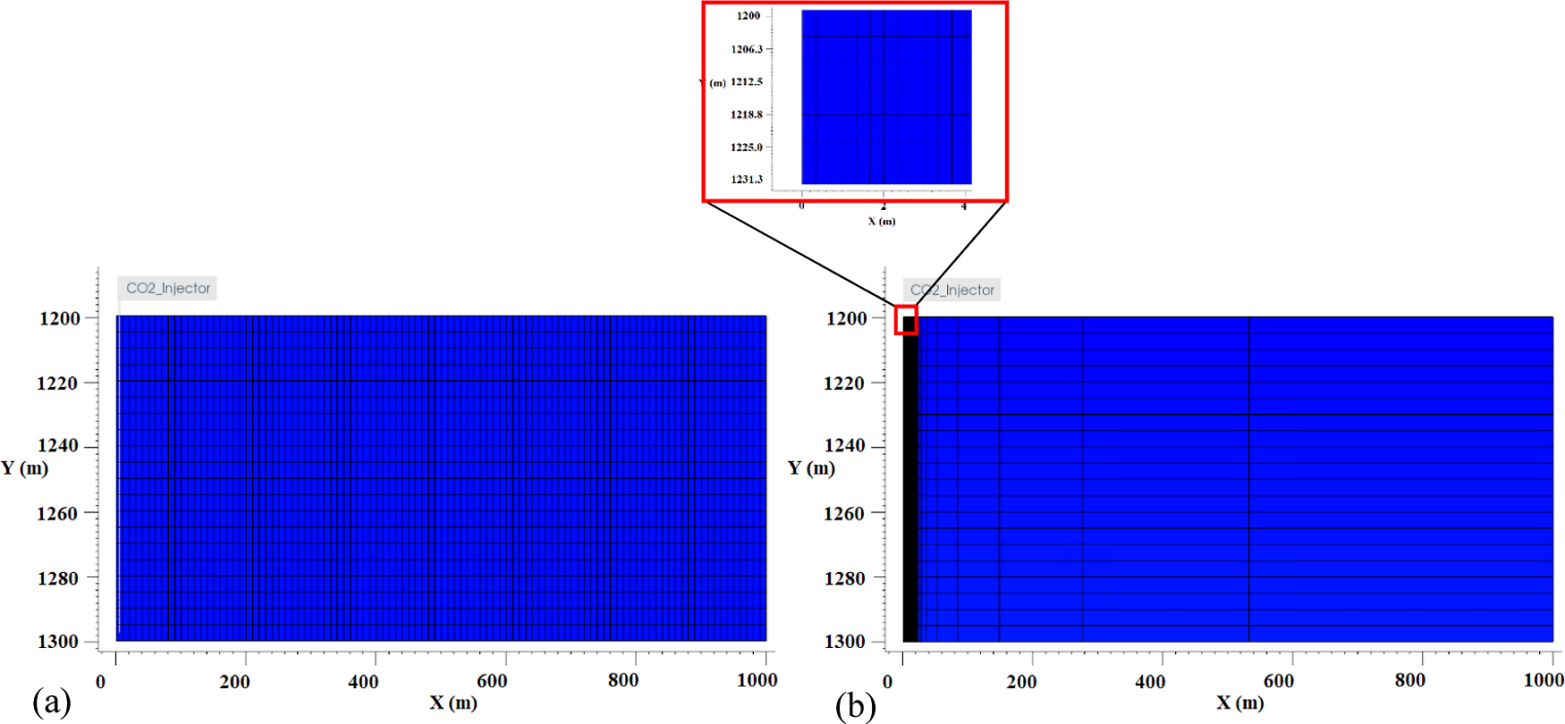
2D simulation model geometry and dimensions; (a) uniform meshing and (b) nonuniform meshing (the first 20 m in the *x*-direction is uniformly refined, and the spacing is doubled beyond that point). CO_2_ is injected into the model from the 3 lower-most left-hand side cells, and injection continues for one year (simulation runs for 200 years).

A two-dimensional rectangular aquifer model of size 1000 m × 100 m is constructed, with two meshing systems: uniform and nonuniform meshing, as shown in Figure 3. In the uniform model (Figure 3a), a uniform grid size of 10 m × 5 m is considered. In the nonuniform model (Figure 3b), a refined uniform grid spacing of 0.33 m in the *x*-direction is used within the first 20 m of the wellbore, doubling beyond this point while maintaining the 5 m spacing in the *y*-direction. This refinement in the near wellbore region is used in scenario 5 to capture the physics of the capillary backflow, which is a near wellbore region phenomenon. The model presents a carbonate reservoir that is fully saturated with brine. The three lower-most cells of the aquifer are perforated near the injection well, located at the left, where CO_2_ is injected into the aquifer. The injection starts in 2024 and continues for one year, and the simulation runs for 200 years to observe the evolution of the CO_2_ plume and its interaction with the subsurface environment. A total amount of 3.66 million m^3^ CO_2_ is injected with an injection rate of 10,000 m^3^/day. The model is assumed to be homogeneous to reduce the degree of complexity for a better understanding of the parameters of interest.

The reservoir conditions and characteristics are summarized in Table 1. Peng–Robinson (PR) equation of state is used for calculating gas properties (density, fugacity, etc.).^68^ Li-Nghiem’s method is used for calculating the Henry’s constant and defining CO_2_ solubility in water.^69^ Kestin and Rowe-Chou models are used for calculating aqueous viscosity and density, respectively.^70,71^

**Table 1:** Reservoir Conditions and Characteristics

temperature	60 °C
permeability	100 md
pressure	13,100 kPa
porosity	0.18
pH	6.0
rock compressibility	5.8 × 10^–7^ 1/kPa
water compressibility	4.5 × 10^–7^ 1/kPa
top of the formation	1200 m

The relative permeability and capillary pressure (Pc) curves are shown in Figure S9 in the Supporting Information.^72^

### Simulation Scenarios

When CO_2_ is injected, it is involved in a number of interactions with the hosting environment that happen at the same time. CO_2_ molecules are dissolved in brine, and water molecules are also mutually dissolved in the CO_2_ stream, leading to water evaporation. Evaporation causes the precipitation of salt, while dissolution of CO_2_ in brine triggers the reaction of brine with rock surfaces as it acidifies the brine through the production of carbonic acid, nurturing the conditions for mineral dissolution and precipitation. As the water is evaporated near the wellbore, a saturation gradient is induced between the near-wellbore region and the inner parts of the aquifer with higher water saturations, producing a capillary gradient that triggers a backflow of brine to the evaporation site as capillary backflow. To investigate the aforementioned mechanisms and phenomena involved in a CO_2_ storage process, a total of 6 scenarios have been considered, as shown in Table 2. These include water evaporation and salt precipitation, CO_2_ dissolution, geochemical reactions, and capillary effects. Another purpose of presenting these scenarios is to provide a background knowledge and a good mechanistic understanding of these interactions that will help to propose other scenarios to tackle the arising challenges from a CO_2_ injection process, such as salt precipitation issue. In each of the scenarios, a different mechanism is targeted, and all of the mechanisms are eventually built up to see the effect of all phenomena on CO_2_ storage. Understanding these interactions individually and collectively is essential to assess their impacts on the process. In scenario 1, only the effect of water evaporation and salt precipitation is considered to provide a baseline for comparison with other scenarios by isolating these fundamental phenomena from other physical and chemical processes. It is crucial, for example, to understand the water evaporation process and how it is integrated with salt precipitation. In the CO_2_ storage processes in aquifers, water evaporation and salt precipitation are two distinct phenomena that work closely together. As the water is dissolved into CO_2_ through evaporation, salt precipitation is triggered, where the solubility limit of salt ions present in the brine can be exceeded, which may lead to salt precipitation.^21^ For this reason, these two phenomena are considered a single scenario in this study. In scenario 2, the effect of the CO_2_ solubility in brine was added to the effect of water evaporation and salt precipitation. By doing so, it will be possible to observe how these phenomena work together and to monitor and explain the role that CO_2_ solubility plays in the overall changes of the system, especially through density-driven convective mixing. The inclusion of CO_2_ solubility in the calculations is crucial, especially for geochemical reactions, as it is the triggering point of these fluid/rock interactions. CO_2_ solubility disrupts the chemical equilibrium in the aqueous phase by producing carbonic acid and increasing the concentration of H^+^ ions, decreasing the pH, and acidifying the environment, leading to geochemical reactions. So, modeling geochemical reactions without including CO_2_ solubility is not possible. Scenarios 3 and 4 consider the phenomena when the effect of mineral dissolution and precipitation has been factored in with consideration of 2 different rock types. Calcite and dolomite have been chosen as the most dominant minerals in carbonates, where their geochemical reaction (dissolution or precipitation) is examined in these scenarios. Extensive research in the literature has shown that the effect of geochemical reactions on porosity change in CO_2_ storage processes may be limited,^73−75^ especially compared to the significant extent of salt precipitation. Using this scenario, it is possible to critically examine these findings, allowing us to decide whether to continue with geochemical reaction effects in the proceeding scenarios. Through scenario 5, the effect of capillary backflow is explained by including the capillary pressure in the simulation. Capillary backflow is triggered by evaporation where the saturation of water near the wellbore is reduced and leads to a saturation gradient across the reservoir that manifests in the form of a counter-current brine flow toward the injection point.^76,77^ This continuous flow of brine provides fresh feed for evaporation near the wellbore and causes significant salt precipitation that may lead to the complete clogging of the well. A sixth scenario was defined to evaluate a proposed remedy to the salt precipitation issue, i.e., dissolved-water CO_2_ injection (dwCO_2_ injection) where CO_2_ is injected with some dissolved contents of water, as an alternative to dry CO_2_ injection. The dissolved water content is expected to reduce the potential for more water evaporation as the CO_2_ stream already contains water; hence, salt precipitation can be prohibited.

**Table 2:** Different Injection Scenarios and Storage Mechanisms and Compositions of Resident Ions, Rock, and Injected CO_2_ in Each Scenario^a^

scenario	water evaporation and salt precipitation	CO_2_ solubility	geochemical reaction	Na^+^ (ppm)	Cl^–^ (ppm)	Ca^2+^ (ppm)	Mg^2+^ (ppm)	rock	injected CO_2_	comments
scenario 1	√	X	X	90,000	135,000			no reactivity	100% CO_2_	
scenario 2	√	√	X	90,000	135,000			no reactivity	100% CO_2_	
scenario 3	√	√	√	90,000	135,000	500		10% calcite	100% CO_2_	calcite rock
scenario 4	√	√	√	90,000	135,000	500	800	95% calcite −5% dolomite	100% CO_2_	calcite/dolomite rock
scenario 5	√	√	X	90,000	135,000			no reactivity	100% CO_2_	including Pc
scenario 6	√	X	X	90,000	135,000			no reactivity	99.37% CO_2_ – 0.63%H_2_O	dissolved-water CO_2_ injection (dwCO_2_ injection)

a Geochemical reaction includes dissolution or precipitation of the carbonate minerals.

The compositions shown in Table 2 are used in different scenarios. Some of these values are from published literature.^78^ In the first, second, and fifth scenarios, in addition to CO_2_ and water, only salt is present in the aquifer (Na^+^ and Cl^–^) to account for the salt precipitation phenomenon. In the third scenario, calcium ion (Ca^2+^) is included because of calcite rock, and in the fourth scenario, magnesium ion (Mg^2+^) is added to the system to account for the dolomite rock. In the last scenario, the CO_2_ stream has been saturated with 0.63% mole fraction (6300 molar ppm) water before injection to the aquifer to perform dissolved-water CO_2_ injection (dwCO_2_ injection).

The reactions shown in Table 3 occur when their respective process is active. While CO_2_ solubility, water evaporation, and salt precipitation are simulated with an equilibrium approach, dolomite and calcite reactions are dealt with by a kinetic approach (CMG default values). In salt precipitation modeling, the ratio of reaction rate to advective rate determines whether a kinetic or equilibrium approach to be used. When these two rates are comparable, a kinetic approach is appropriate, but when the advective rate is lower, an equilibrium approach can be adopted.^79,80^ Because of a low advective rate, an equilibrium approach is selected in this study (10,000 m^3^/day injection rate compared to some hundred thousand m^3^/day field-scale injection rates). Adopting a kinetic approach would result in a more distributed salt precipitation pattern in the porous medium; however, it would still be restricted to the equilibrium assumption implemented in the evaporation model.^81,82^

**Table 3:** List of Reactions^a^

reaction name	formulas
water evaporation	H_2_O (aq) ⇔ H_2_O (g)
CO_2_ solubility	CO_2_ (g) ⇔ CO_2_ (aq)
water–carbonation	H_2_O (aq) + CO_2_ (aq) ⇔ H_2_CO_3_ ⇔ H^+^ + HCO_3_^–^
bicarbonate dissociation	HCO_3_^–^ ⇔ H^+^ + CO_3_^–2^
water dissociation	H_2_O (aq) ⇔ H^+^ + OH^–^
salt precipitation	NaCl (s) ⇔ Na^+^ + Cl^–^
calcite reaction	CaCO_3_ (s) + H^+^ ⇔ Ca^2+^ + HCO_3_^–^
dolomite reaction	CaMg(CO_3_)_2_ (s) + 2H^+^ ⇔ Ca^2+^ + 2HCO_3_^–^ + Mg^2+^

a CO_2_ is dissolved into the brine and creates carbonic acid, which decomposes into its constituents and increases the acidity of brine. This acid, then, reacts with rocks (calcite and dolomite) and causes rock dissolution and precipitation. Water is also evaporated into the CO_2_ phase and causes salt precipitation.

## Results

This section discusses the results of the different scenarios mentioned above. After demonstrating the mechanisms of all five phenomena (water evaporation, salt precipitation, CO_2_ dissolution, geochemical reactions, and capillary backflow) through the first five scenarios, the sixth scenario focuses on showing the effect of dissolved-water CO_2_ injection (dwCO_2_ injection) on preventing salt precipitation. The challenges and feasibility of this proposed methodology have been discussed in the Supporting Information (Theoretical Background), and a real example of a CCS project is included in this section that gives an analysis of the dissolved-water CO_2_ injection (dwCO_2_ injection).

### Scenario 1: Water Evaporation and Salt Precipitation

In this scenario, only water evaporation and salt precipitation are considered. The mineral reactions were excluded, and the CO_2_ solubility option was also turned off by directly modifying the project’s data file as this option is always active by default in the software. By doing so, water mole fraction reduction in brine solution will be a good indicator of water evaporation. This is because, when the CO_2_ solubility is off and reactions are also excluded in this scenario, there will be no other source that would reduce the water mole fraction other than evaporation.

**Figure 4:**
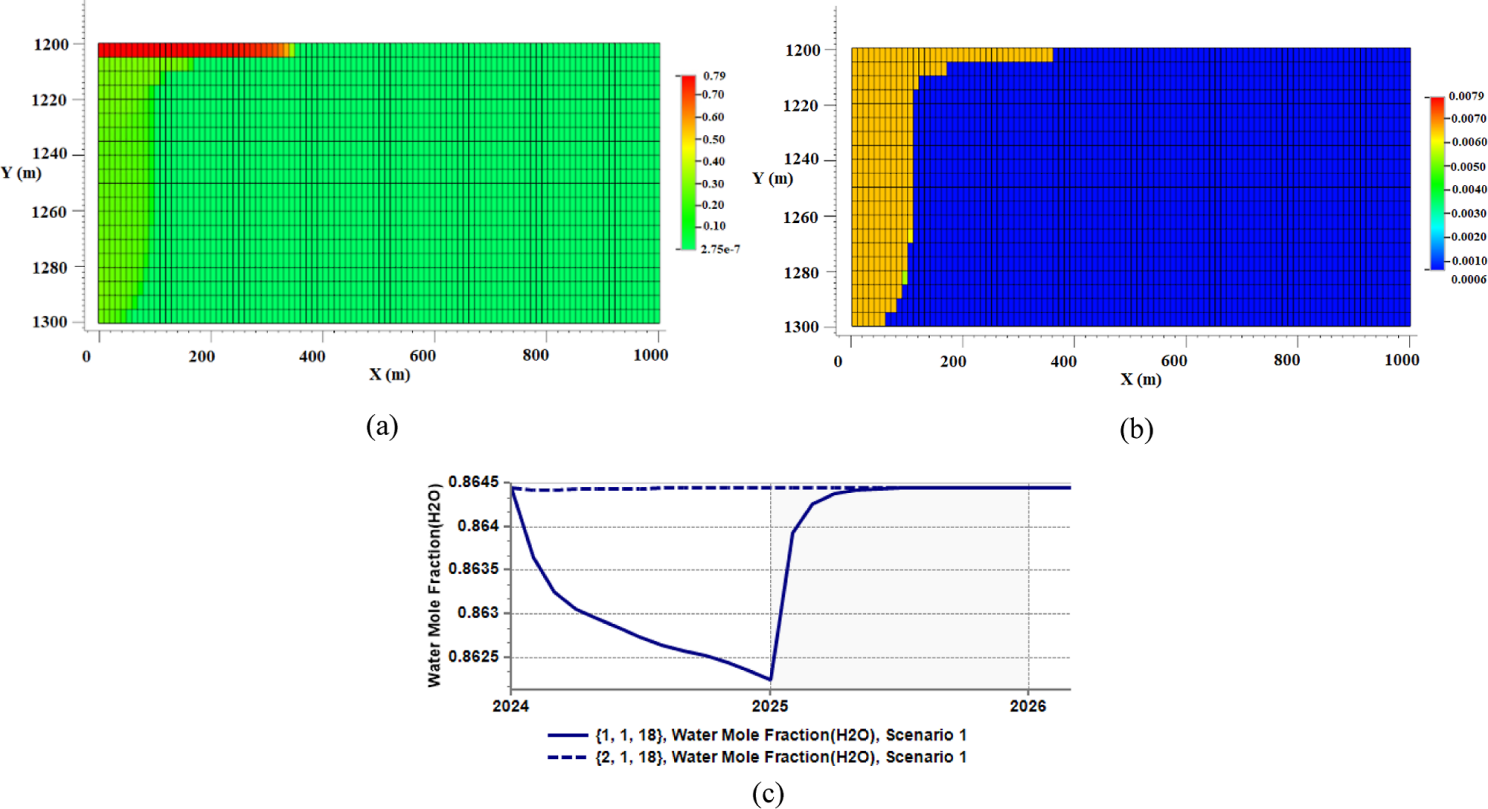
Results of scenario 1 (water evaporation and salt precipitation only) at the end of the simulation. (a) Gas saturation profile; gas overrides brine and is trapped under the caprock, (b) water mole fraction in the gas phase; a uniform gas water content at equilibrium with liquid is resulted, and (c) water mole fraction in the liquid phase at an inlet cell (solid line) and its neighboring block (dashed line); evaporation occurs only at the inlet cell.

**Figure 5:**
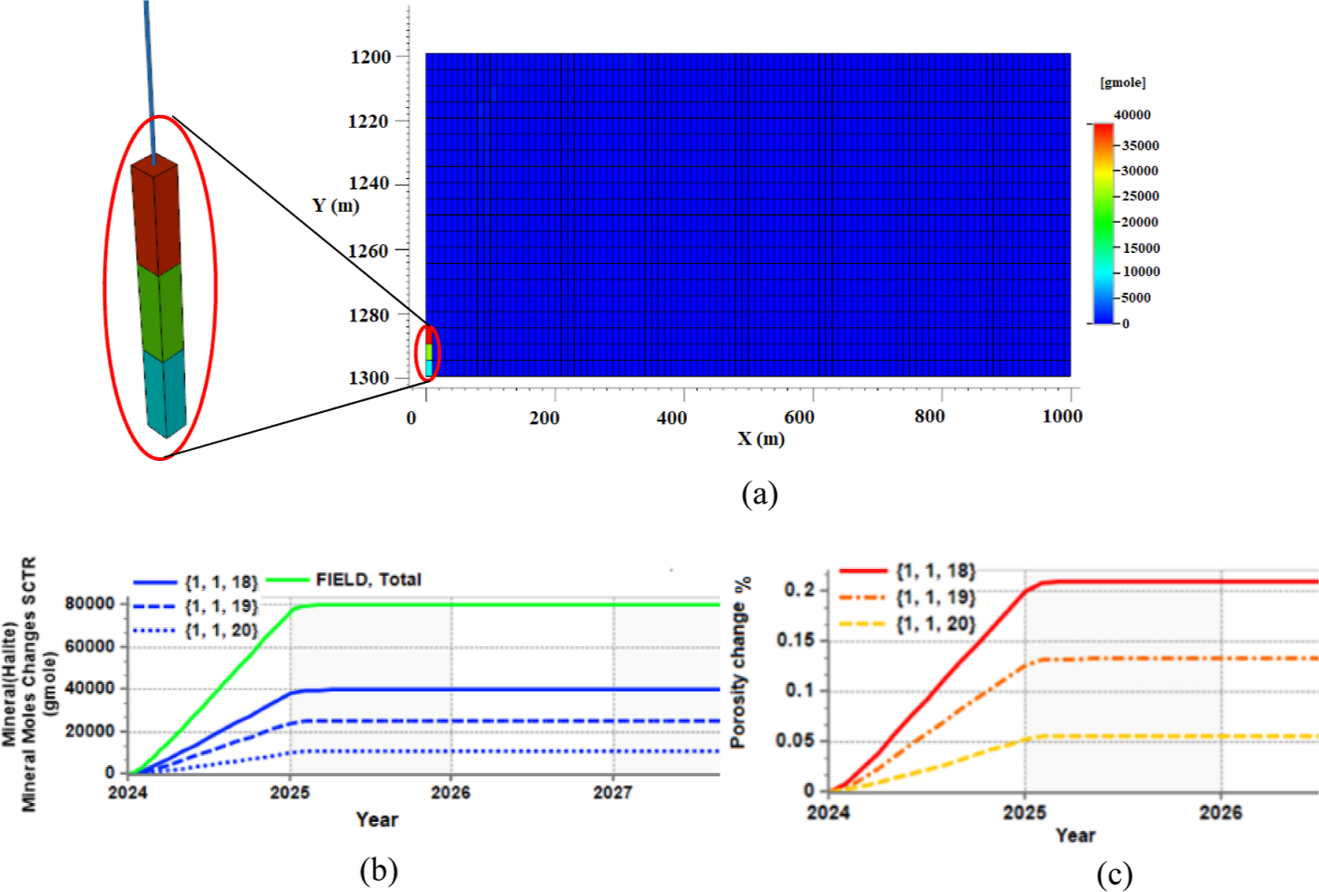
Results of scenario 1 (water evaporation and salt precipitation only) at the end of the simulation. (a) Salt precipitation profile in the aquifer; significant amount of salt precipitation happens at the inlet, (b) total amount of precipitated salt and its breakdown at each injection point, and (c) porosity reduction in each injection points due to salt precipitation; the upper cell experiences more salt precipitation and damage due to gravity effects.

After CO_2_ is injected, brine is displaced from around the well. Brine displacement reduces the water saturation in affected areas. This can be seen from the saturation map in Figure 4a that shows an increase in gas saturation. This gas saturation change also shows that gas has overridden brine and has traveled to the top of the aquifer at the end of the simulation, which confirms structural trapping. Apart from these, water evaporation occurs at the inlet cell. This can be inferred through the reduction of the water mole fraction of the liquid phase in the inlet cells, as evident from Figure 4c (solid line) that shows the water mole fraction profile of the cell (1 1 18). The evaporation stops as the CO_2_ injection is stopped after one year. This trend is the same in the other two cells of the CO_2_ inlet. As an equilibrium approach is assumed for the evaporation process (i.e., equal fugacities of water components in the aqueous and gaseous phases at every thermodynamic condition), the gas phase reaches its maximum water content at that thermodynamic condition after its first touch with the resident brine right at the reservoir inlet. So, water-saturated CO_2_ travels from that point of contact (the injection point) toward the interior of the aquifer. As this migration happens through the aquifer, the water content of gas (the molar fraction of water dissolved in the gas phase) jumps to a nearly uniform value at the points swept by the gas phase, as shown in Figure 4b. This saturation limit is almost 0.0065 in water molar fraction in this simulation case. Therefore, because the migrating CO_2_ is almost fully saturated by water, it almost does not vaporize any more water as it moves further in the aquifer. This can be seen from Figure 4c (dashed line), which shows the amount of water mole fraction of the liquid phase in the cell (2 1 18), the neighboring cell of the inlet cell (1 1 18). Water mole fraction in this cell stays almost the same, as opposed to that of the inlet cell, which is a sign of no evaporation (an infinitesimally slight increase happens to the water content in the gas phase here that cannot be seen in Figure 4c, which signifies a slight evaporation at the neighboring block due to the changes in thermodynamic conditions). Similarly, because an equilibrium approach is assumed for salt reaction (i.e., its components are at the equilibrium conditions), salt precipitation also occurs at its solubility limit as water is evaporated. Hence, three phenomena happen at the same time: water displacement, water evaporation, and salt precipitation. As the majority of water evaporation happens right at the inlet cell, a substantial amount of salt precipitation happens at this point of the aquifer, as shown by Figure 5a. As soon as the injection of fresh CO_2_ is stopped, evaporation and salt precipitation also stop. At the end of the injection, 80,000 g mol salt is precipitated in the aquifer: 40,000 g mol at the upper injection cell (1 1 18), 25,000 g mol at the middle injection cell (1 1 19), and 10,000 g mol at lower injection cell (1 1 20) (infinitesimal precipitations in the order of 6 decimals of porosity reduction also happen at other cells that have been swept by the gas phase but cannot be spotted in the figure). These precipitations, respectively, translate into 0.21%, 0.13%, and 0.05% porosity reduction in these three injection cells after one year of CO_2_ injection—these are shown in Figure 5b,c. Similar results have been reported in the literature with overall porosity loss of around 0.2% to 0.38% at high injection rates, which are equivalent to the case of neglecting capillary effects in this scenario.^83^

### Scenario 2: Water Evaporation, Salt Precipitation, and CO_2_ Dissolution

**Figure 6:**
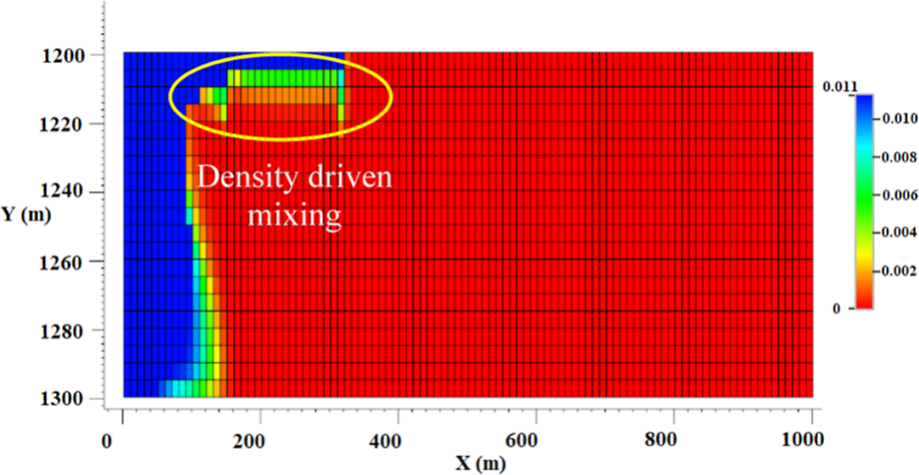
Scenario 2, CO_2_ solubility effect; mole fraction of CO_2_ in the liquid phase; as the value of dissolved CO_2_ in the brine phase increases, the liquid starts to fall off the top of the aquifer and density-driven convective mixing happens (the area marked by a yellow oval).

By activating the gas solubility option in simulations, this scenario allows for the dissolution of CO_2_ in the brine solution (Figure 6). How much CO_2_ will dissolve in brine will depend on its solubility limit, which is a function of salinity, pressure, and temperature. As soon as the CO_2_ is injected, it reaches its solubility limit in the brine phase in thermodynamic conditions based on the equilibrium approach (equal CO_2_ fugacities in the liquid and gas phases). While CO_2_ is dissolving in brine, water molecules evaporate into the gas phase, salt precipitates, and brine is displaced with the same principles as before. These phenomena happen at the same time at the injection point at every time step where fresh CO_2_ is injected into the model. So, the outflow of an injection cell is a CO_2_-saturated brine solution and a water-saturated gas stream. The compositions of these two components (CO_2_ in brine and water in gas) stay almost the same during the rest of the simulation since they are at their solubility limit. By the dissolution of CO_2_ in brine, a carbonic acid solution is formed that has a density higher than that of brine. As the CO_2_ travels to the top of the aquifer, the liquid phase at that point gets denser due to CO_2_ dissolution, and a point is reached where the carbonated brine becomes heavier than the resident brine phase below it and starts to flow downward. So, density-driven convective mixing happens, as shown in Figure 6 (the yellow oval). The composition of CO_2_ in the liquid phase is around 0.01. If the slight reduction to salt precipitation in this case is neglected, it can be said that all of the other mechanisms of the process are similar to the last case (fluid flow and species transport, salt precipitation, porosity reduction, etc.).

### Scenarios 3 and 4: Water Evaporation, Salt Precipitation, CO_2_ Dissolution, and Geochemical Reactions

The difference in these scenarios compared to before is the inclusion of rock geochemistry; hence, the salt precipitation and mutual CO_2_/water solubility principles stay the same as previous scenarios with the adoption of an equilibrium approach. When CO_2_ is dissolved in water, it reacts with water molecules in the water–carbonation reaction and creates more H^+^ and HCO_3_^–^ ions. This leads to changes in pH and increased acidity of the aqueous phase that can cause mineral dissolution. Changes in the aqueous ions’ concentration disrupt the liquid/rock equilibrium that causes dissolution or precipitation of the mineral depending on the new equilibrium conditions. This is defined by the saturation ratio and saturation index. Saturation ratio is defined as the ratio of the solution’s ionic activity product to its equilibrium constant. Saturation index of minerals is defined as the logarithm of saturation ratio and is a measure of the distance from equilibrium.^84^ If the saturation index is positive, the solution is supersaturated, and the reaction goes toward the precipitation side. If it is negative, the mineral dissolves in the brine solution, and the reaction goes toward the side where ions’ concentration can increase. Because the amount of dissolved CO_2_ in brine solution is limited to its solubility limit of that governing thermodynamic condition, the dissolution or precipitation of minerals will also stay limited (this has been discussed in Supporting Information in details). Reactive surface area and the evolution of the porous geometry are accounted for internally in the software in the reaction kinetic model. If a kinetic approach was adopted for salt precipitation, the related mineral reaction would also be a function of reactive surface area and the distance from equilibrium.^85−87^ However, due to an equilibrium approach and the continuous evaporation of water, salt precipitation can continue until full dry-out of the system (where all the water is evaporated). Hence, the extents of these two phenomena are not on the same scale. Geochemical reaction is limited to its equilibrium condition, and after the acid is spent, no more reaction happens, but salt precipitation can happen limitlessly. Hence, geochemical reaction does not tremendously contribute to porosity change as opposed to salt precipitation.^88^ As also mentioned before, CO_2_ solubility is a function of salinity, in addition to pressure and temperature.^89^ If the salinity of brine is lowered, higher values of CO_2_ can dissolve in water, so a lower pH and higher acidity resulted from that carbonated brine. Therefore, one way to increase liquid/rock interactions or dissolution/precipitation patterns is to change the brine salinity.

**Figure 7:**
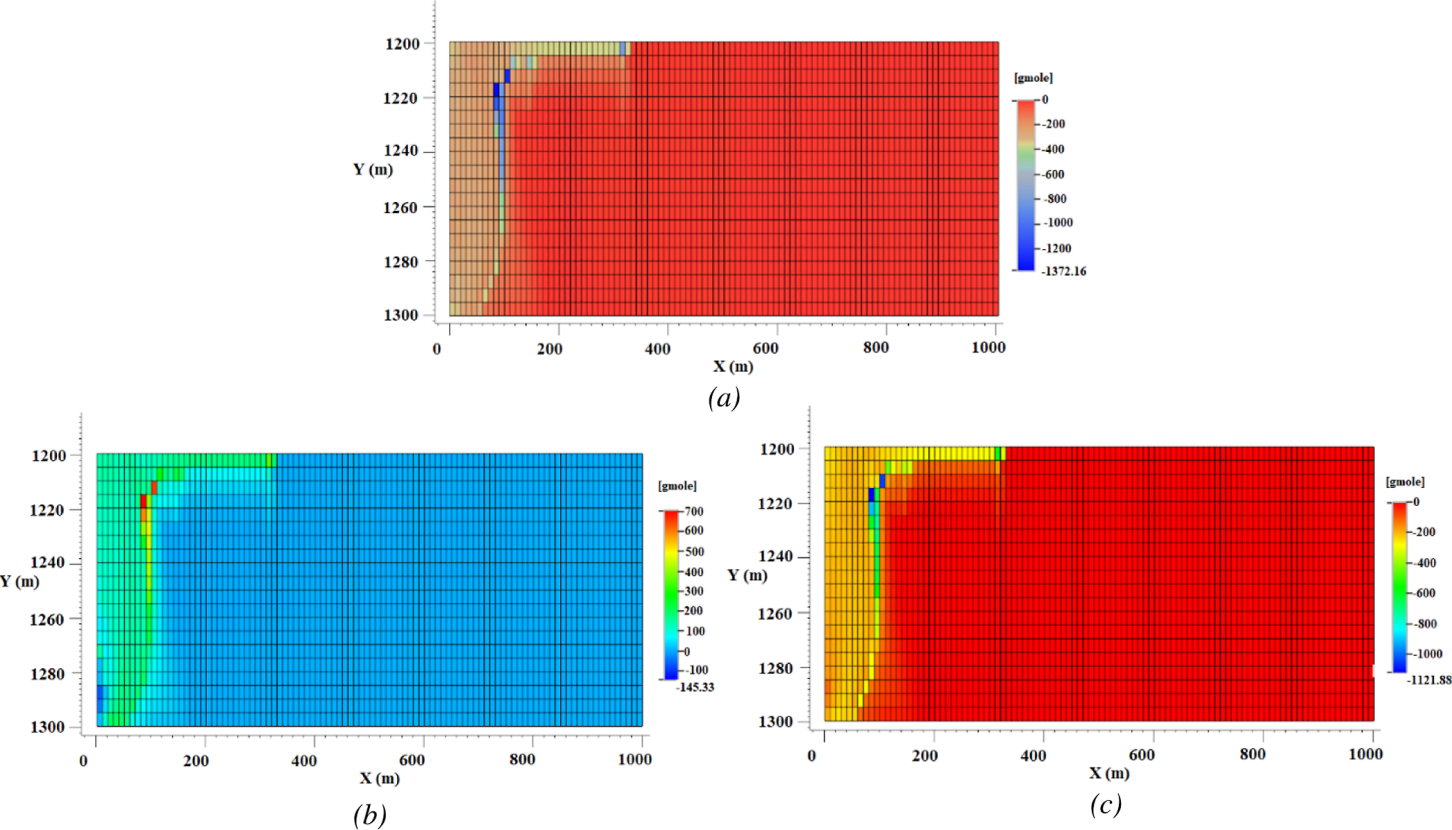
Amount of (a) dissolved calcite in scenario 3, (b) precipitated calcite in scenario 4, and (c) dissolved dolomite in scenario 4; in the presence of dolomite, calcite precipitates, but in its absence, it dissolves. Also, the extent of rock dissolution and precipitation stays limited to an equilibrium condition.

In scenario 3, a calcite reactive rock is considered (10%). In scenario 4, a rock composed of 95% calcite and 5% dolomite is considered. In the presence of dolomite, calcite precipitates, but in its absence, it dissolves (negative change). The rock dissolution pattern of scenario 3 is shown in Figure 7a, and the rock dissolution/precipitation patterns of scenario 4 are shown in Figure 7b,c. As can be seen from the reactions of Table 3, there is a competition between calcite and dolomite over the consumption of the H^+^ cation. This is the reason for different dissolution/precipitation behavior of calcite in the two cases as dolomite wins the competition over calcite in scenario 4 and dissolves by consuming H^+^ cations because the amount of magnesium is small (undersaturated). This forces the calcite to precipitate by supersaturation of calcium ions in the solution. Different calcite–dolomite dissolution/precipitation patterns have been reported in the literature, mainly because of the different governing conditions such as the presence of other minerals and the difference in the ions’ concentrations. Simulation results of the injection of CO_2_ into a Middle Eastern carbonate rock have shown that both calcite and dolomite dissolve in the system. This was mainly because of the presence of other ions (such as Na, Cl, K, Ca, Mg, Sr, Fe, C, and S) with concentrations that nurture an environment for carbonate dissolution.^74^ On the other hand, a dissolved calcite/precipitated dolomite pattern has also been reported in the simulation of CO_2_ injection into the Permian basin of western Texas with a carbonate rock (containing calcite, dolomite, ankerite, anhydrite, and negligible amounts of quartz, illite, and kaolinite) and different dissolved ions in the brine (such as Na, Cl, K, Ca, Mg, Al, and Fe). These conditions, especially the reservoir temperature and supersaturated concentration of magnesium, led to the precipitation of dolomite.^13^ By comparing the carbon mineralization patterns of Figure 7 with brine flow pathways of Figure 6, traces of mineral dissolution/precipitation can be spotted along the convection pathways, which shows geochemical reaction is happening at those points. This means that density-driven convective mixing can help carbon mineralization. Convective mixing can transport the CO_2_ to locations at the bottom of the formation where access was difficult due to the CO_2_ gravity override and low sweep efficiency. By dissolution of CO_2_ into the brine and its transportation through convective mixing, more rock is exposed to fresh acid, and this boosts mineralization trapping.

### Cumulative Effect of Geochemical Reactions and Salt Precipitation on Porosity

Porosity change due to mineral changes (salt and rock) stays almost the same as in previous scenarios, meaning that there is no difference between the scenario of salt precipitation alone and when there is calcite or calcite/dolomite as well. Calcite dissolution in each cell in scenario 3 is in the range of 0–1372 mol, which is less than 2.7 mol/m^3^ of the reservoir rock (cell volumes 500 m^3^). Calcite precipitation and dolomite dissolution in each cell in scenario 4 are in the ranges of 0–709 mol (less than 1.4 mol/m^3^ of the reservoir rock) and 0–1122 mol (less than 2.2 mol/m^3^ of the reservoir rock), respectively. The average dissolution value of calcite in scenario 3 and dolomite in scenario 4 is both around 0.5 mol/m^3^ of the reservoir rock, and the average value of calcite precipitation in scenario 4 is around 0.25 mol/m^3^ of the reservoir rock. Porosity changes due to these average dissolution and precipitation values are **+1.8 × 10**^**–5**^ for calcite dissolution in scenario 3 and **+2.3 × 10**^**–5**^ for scenario 4 (net summation of +3.2 × 10^–5^ porosity change due to dolomite dissolution and −9 × 10^–6^ porosity change due to calcite precipitation). As can be seen, the extent of geochemical reaction on porosity change is very limited (in order of 5 decimals, around ∼0.002% of rock). This means that the new porosity due to mineralization will be around 18.002%, which is a very negligible change. These are consistent with values reported in the literature, where the amount of dissolved or precipitated minerals in carbonates is shown to be limited, in the order of 0.1 to 10 mol per cubic meter of reservoir rock, equivalent to 4 to 6 decimals of rock volume change.^13,73−75,88^ Less than 5 mol calcite and 1 mol dolomite per reservoir cubic meter have been reported to be dissolved from the simulation results of CO_2_ injection into a Middle Eastern carbonate rock, with a maximum porosity change of +6 × 10^–4^, which is equal to only 0.06% porosity increase.^74^ On average, around 0.2 kg/m^3^ dissolved calcite and 0.1 kg/m^3^ precipitated dolomite have been resulted from the simulation of CO_2_ injection into the Permian basin of western Texas.^13^ This is, respectively, equivalent to a rock volume change of +7.4 × 10^–5^ and −3.5 × 10^–5^ per reservoir volume, bringing the average total porosity change due to calcite and dolomite geochemical reactions to around 0.004%, which is a negligible amount. These have been discussed in more detail in the Supporting Information. In geochemical reactions, the amount of mineral dissolution and precipitation can only reach its equilibrium value, and after that, the reaction stops. On the other hand, in the salt precipitation, an instantaneous equilibrium is governing the process, meaning that salt is at its solubility limit, and the whole excess salt can precipitate with any changes of the molalities. That is why in these two scenarios, salt precipitation dominates, and the porosity change due to geochemical reaction does not significantly alter the porosity compared to previous scenarios.

### Scenario 5: Capillary Pressure and Capillary Backflow Effects

**Figure 8:**
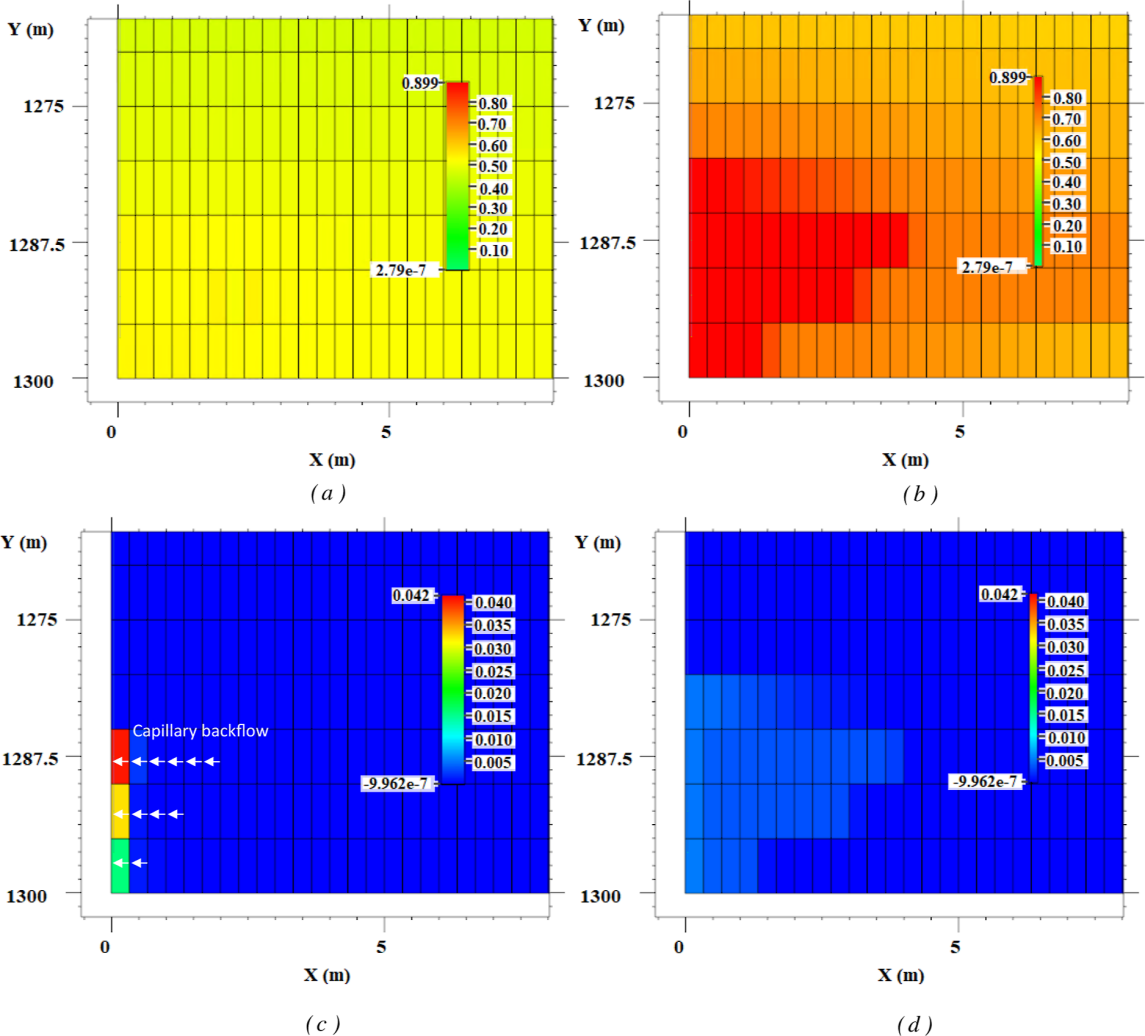
Effect of capillary backflow: (a) gas saturation in scenario 5, (b) gas saturation in scenario 2, (c) porosity reduction due to salt precipitation in scenario 5, and (d) porosity reduction due to salt precipitation in scenario 2; without capillary pressure, a dry-out front propagates in the vertical and horizontal direction due to domination of viscous and gravity forces that lead to a uniform salt precipitation pattern. In the presence of capillary forces, capillary forces dominate the system, and localized salt precipitation happens near the wellbore due to capillary backflow.

In this scenario, capillary pressure is included in simulations to see how the capillary backflow phenomenon affects salt precipitation. As the effect of geochemistry was proven to be limited compared to salt precipitation in the last scenario, geochemistry is excluded from this scenario. The results of this scenario are compared with a similar case without capillary pressure (scenario 2 with the refined mesh) and are shown in Figure 8, where the area near the wellbore at the end of the injection is illustrated. The aspect ratio of the figures is scaled to depict the near-wellbore region. The gas saturation distribution of the two scenarios is shown in Figure 8a,b. A uniform gas saturation (around 50%) results from the CO_2_ injection model that includes capillary pressure effects, as shown in Figure 8a. In contrast, a dry-out front is formed near the injection zone in the scenario without capillary effects, as demonstrated by Figure 8b, where the residual brine is evaporated (the water saturation threshold for water evaporation was set to 0.1). The distinct behavior observed between the two cases arises from the competing effects of various forces at play. When the capillary forces are not accounted for, viscous and gravitational forces dominate, which lead to the progression of the CO_2_ dry-out front. However, capillary forces, when they are accounted for, oppose viscous and gravitational forces. This resisting force results in a uniform saturation distribution in the system, where the dry-out front is held back by capillary forces. These results are consistent with experimental observations in the literature.^24,90^ It has been reported in the literature that injecting CO_2_ in a high-capillary brine-saturated rock such as a shale would lead to a more uniform brine saturation distribution in the model than in the case of a low-capillary reservoir rock.^24^ Also, other observations have proved the existence of a resistance period against water dry-out dominance due to the effect of capillary forces.^90^

The capillary forces equilibrate the saturation across porous medium, and the capillary backflow transports brine to the near-wellbore area. The principle of capillary backflow is based on the evaporation-induced capillary pressure disequilibrium caused by the saturation gradient produced in the system between the low-saturation near-wellbore region and the highly saturated inner parts of the aquifer. The brine backflow occurs in response to evaporation, attempting to re-equilibrate the system by redistributing the brine saturation in the model via counter-current imbibition to the near-wellbore region, compensating for the amount of water saturation lost by evaporation.^21^

Capillary backflow leads to an adverse effect on salt precipitation upon intensifying it. Figure 8c,d demonstrates the porosity change due to salt precipitation in the two scenarios under investigation (scenarios 2 and 5). As evident from Figure 8c, significant localized salt accumulation occurs in the scenario with capillary forces (scenario 5), while minor salt precipitation occurs in the scenario without capillary effects (scenario 2, Figure 8d). The amount of precipitated salt in the upper injection cells in scenario 5 exceeds two-fold the salt originally in place, which confirms the occurrence of capillary backflow phenomenon. When capillary effects are neglected, the dry-out front propagates deeper into the aquifer, and salt precipitation only happens in situ, resulting from the evaporation of trapped brine patches, as also observed in past experiments.^65,66^ In this scenario, salt precipitation is uniformly distributed over a length scale of the near-wellbore area, with a damaged zone extending up to 4 m. Consequently, salt accumulation remains minor, leading to a porosity change of only 0.2–0.5%, with negligible permeability and injectivity reduction. This aligns well with the experimental observations in the literature, showing that in the absence of capillary backflow, an extensive salt coverage in the form of a surface coating with minor impairment is resulted from the evaporation of brine during CO_2_ injection.^21^ It is worth noting that in scenario 2, the precipitation pattern superimposes with its gas distribution, which reaffirms salt precipitation in the dry-out zone, and that the process is both viscous- and gravity-dominated, resulting in a salt pattern distributed in both longitudinal and transversal directions to the well location. In contrast, scenario 5 is a fully capillary-dominated process due to the effect of the capillary pressure. As can be seen from Figure 8c, the dry-out front does not propagate in the system, and salt is only precipitated at the immediate location near the inlet in this scenario, with no longitudinal or lateral extension. Due to the dominance of capillary forces over viscous and gravity forces, capillary backflow continuously transports brine as a feed to the evaporation point, and this causes localized salt accumulation in the region near the wellbore, resulting in a significant damage compared to scenario 2. The porosity reduction due to this salt precipitation in the upper, middle, and lower cells near the perforation is, respectively, equal to 4.1%, 3.2%, and 1.6%, showing an increase of up to around 10-fold with respect to scenario 2. Based on the Kozeny–Carman equation,^91^ these are equivalent to 13.6%, 10.7%, and 5.5% permeability reduction in those cells, which are significant for this one-year injection scenario. Over a 30 to 40 years injection lifespan, this may lead to full clogging of the injection interval.

### Proposing a Solution to Salt Precipitation—Scenario 6: Dissolved-Water CO_2_ Injection

In the previous scenarios, the mechanisms of a CO_2_ storage process were discussed. It was shown how the water molecules evaporate into the gas phase, the process through which CO_2_ molecules dissolved in liquid was demonstrated, and the principles behind salt precipitation, geochemical reaction, and capillary backflow were assessed. Analyzing all mechanisms involved in a CO_2_ storage process suggested a new solution to tackle salt precipitation. As was shown, salt precipitation happens due to the evaporation of water molecules into the gas phase until the CO_2_ stream becomes saturated with water. Therefore, it is suggested here that if the injected gas phase is already saturated with the water molecules at the time of injection, this prevents evaporation of more water molecules and, hence, prevents salt precipitations. To evaluate this proposed theory, a case scenario was created based on the insights that were achieved through previous scenarios. In this scenario, it was decided to saturate the injected CO_2_ with 0.63% water content to perform dissolved-water CO_2_ injection (dwCO_2_ injection). The logic for choosing this composition was based on the saturation limit of water in the gas phase at the equilibrium conditions of this study, which was achieved through previous scenarios. This means that injection in this scenario can also be considered a water-saturated CO_2_ injection (wsCO_2_ injection) process. A case with a 0.2% water content is also considered. In this method, less than 1% water content is present in the CO_2_ stream; therefore, the efficiency of the storage process would not be affected compared to other water-based methods such as water-alternating-gas injection.^92−94^ In order to avoid complexities in this simulation, CO_2_ solubility, geochemical reaction, and capillary effects have been neglected to stay focused on the process of water evaporation and salt precipitation.

**Figure 9:**
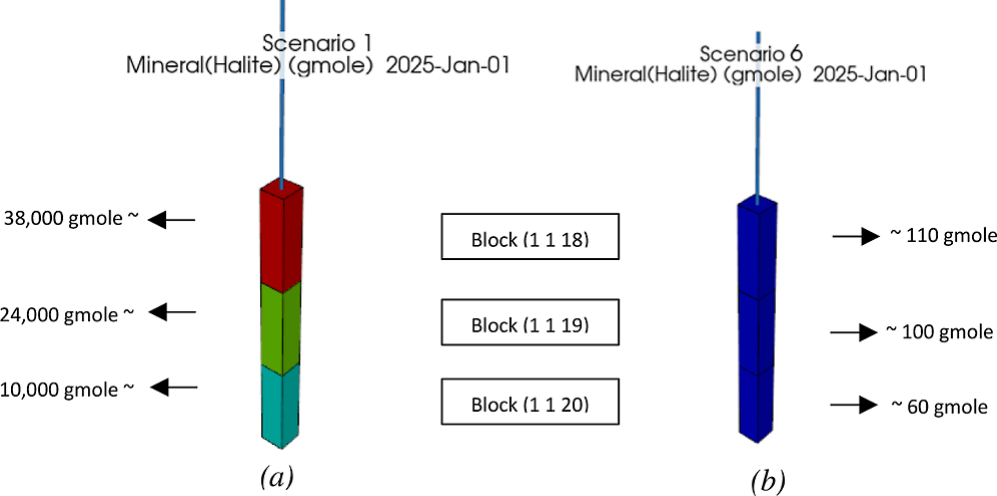
Comparison of the precipitated salt in (a) scenario 1 (dry injection) and (b) scenario 6 (dwCO_2_ injection) at the injection cells; salt precipitation is significantly reduced using the dwCO_2_ injection method.

The results of this scenario are shown and compared with a corresponding scenario (scenario 1) in Figure 9. As can be seen, compared to scenario 1 (dry injection—Figure 9a), salt precipitation dramatically decreases in the new scenario (dwCO_2_ injection—Figure 9b). One can neglect the salt precipitation in this dwCO_2_ injection scenario, as the corresponding porosity change due to this case is infinitesimal. In another simulation, injecting CO_2_ with 2000 ppm molar fraction of dissolved water content (0.2% molar fraction water content) was shown to reduce salt precipitation by one-third. These results confirm the efficacy of the proposed method in preventing salt precipitation.

These results show the technicality of the proposed solution, and the practicality of the approach depends on the choice of the injection method. A sensitivity analysis shows that the proportions of the water saturation value present in the CO_2_ phase are linearly correlated with the percentage of salt precipitation reduction. This means that a 300 ppmv water content in the CO_2_ phase (5% of the saturation value) can result in a 5% reduction in salt precipitation. Based on this analysis, at low recommended water contents (30, 50, or 70 ppmv as practiced in projects such as Northern Lights project,^40^ Porthos project,^95^ etc.), salt precipitation reduction is negligible (around 1%). If the first method of injection (mixing within the well) is chosen, there will be almost no restriction on the amount of dissolved water content, and the CO_2_ phase may be fully saturated with water. As the CO_2_ phase reaches the bottom of the well, it approaches reservoir conditions, and water contents closer to saturation point can be dissolved in CO_2_. Hence, salt precipitation can be fully stopped. As extra safety measures, hydrate inhibitors may be used, and the bottom leg of the well can be completed with appropriate well materials. The amount of water content in the second injection method, however, is restricted to the maximum allowable water content in the CO_2_ phase without free water formation at the surface to avoid corrosion risks in pipes and other infrastructures. As mentioned in the Introduction, this value needs to be engineered as it depends on the thermodynamic conditions of the injection and is highly operational-dependent.^32,50,96,97^ Based on Figure 2, this tolerable amount at the reservoir pressure and temperature conditions of this study (60 °C and 120 bar) is equal to 8500 ppmv. This means that there is a safe window of more than 2000 ppmv of water between the tolerable limit and the current composition of water in this scenario. Based on this figure, even if the surface injection temperature, depending on the winter- or summer-time weather conditions, is reduced to 15–25 °C, still 2500–3500 ppmv water content can be tolerated in the CO_2_ stream without any risk of damage to the infrastructure, and a 2000 ppmv water content can reduce the salt precipitation to one-third. A more detailed analysis of the tolerable water limit is given in the Supporting Information. The next paragraphs present a real case example of the Gorgon CCS project that shows dissolved-water CO_2_ injection (dwCO_2_ injection) has been practiced in the industry with the use of the second injection method (saturation at surface). The example presented here serves to emphasize the importance of proper engineering and design and discusses the root cause of the process failure and how such issues can be mitigated in the future.

**Figure 10:**
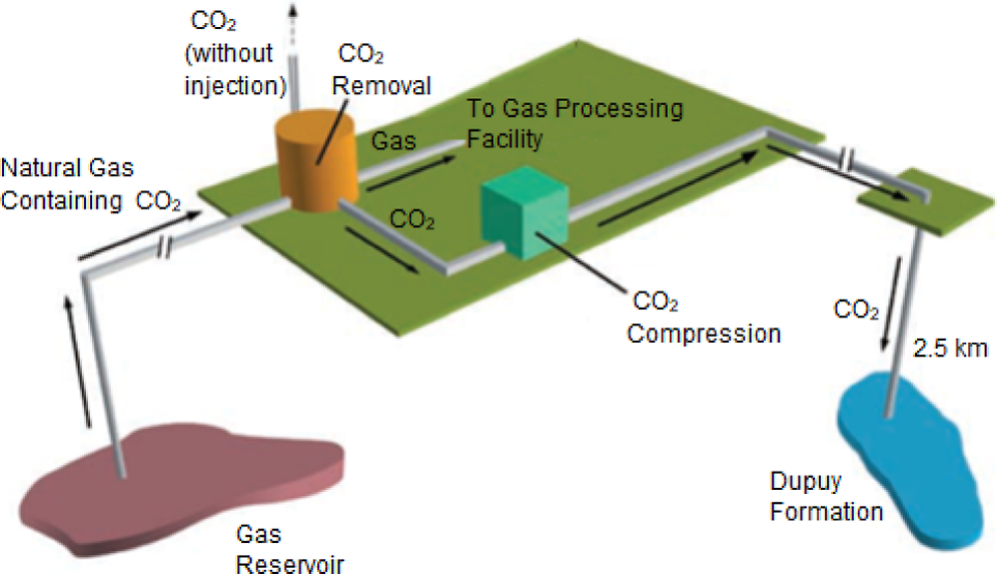
Schematic of Gorgon CCS project; after capturing CO_2_ from natural gas, it is compressed and transported for storage. Reproduced from ref (99). Copyright 2017 Peter Milne The West Australian.

Gorgon CCS project: there is little information about CCS projects but, to the best of our knowledge, there is at least one industrial CCS project that has attempted to conduct dissolved-water CO_2_ injection—Gorgon CCS project.^98^ The incentive here was not to prevent salt precipitation but rather was driven by economic considerations. In this project, the dehydration unit was removed to cut some of the expenses as the water content of the CO_2_-rich phase could stay dissolved within the supercritical CO_2_, and the operational condition was away from free water dropout. Figure 10 demonstrates the schematic of this onshore project.^99^ It shows the stages that the CO_2_ stream undergoes from production in a gas field, separation in a CO_2_ removal unit, to compression and transportation via pipelines to the injection site for injection into the subsurface. In standard CCS processes, a dehydration stage follows CO_2_ compression to remove moisture from the CO_2_ before it is transported through pipelines. Depending on the offshore or onshore nature of the project, different pipeline facilities may be used. If the process is offshore, an additional trunk line is provided to transport the CO_2_ along the seabed to the wellhead. In contrast, for onshore processes, the seabed trunk line does not exist, and the wellhead operation occurs at surface conditions. As mentioned, the Gorgon CCS project is an onshore injection process.^53^

**Figure 11:**
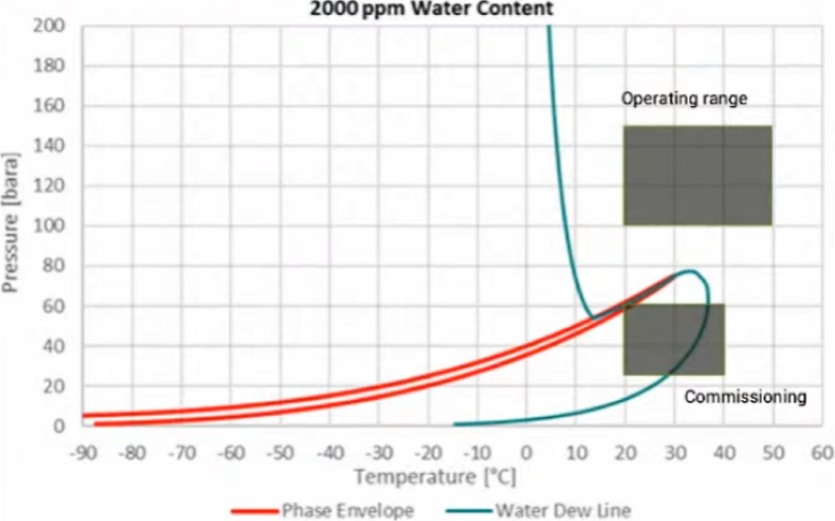
Phase diagram and different operating conditions of Gorgon CCS project. Reproduced from ref (100). Available under a CC-BY-SA license. Copyright 2023 Society of Petroleum Engineers.

The phase diagram and different operating conditions of this CCS project are shown in Figure 11.^100^ The red curve shows the phase envelope of CO_2_-rich phase, and the blue line shows the water dew line when the dissolved water content of CO_2_ is 2000 ppm. Water stays dissolved in the CO_2_ phase when the storage process operates in the region to the right-hand side of the dew curve, but free water is formed in the system if the operation is performed in the left-hand side area of this blue line. The upper shaded rectangle shows the normal operating condition of this project. As can be seen, this range lies within supercritical condition to the right side of the dew curve, and over 2000 ppm of water can be dissolved in the CO_2_ dense phase without water dropout. Therefore, in this project, it was decided to directly inject the water-saturated CO_2_ that comes from the CO_2_ capture unit into the aquifer without dehydrating it, and hence, the dehydration unit was not installed.

However, the project failed due to an engineering error that occurred during the process design as the process engineers did not account for all of the operating conditions of their injection process. During the commissioning, which is a transient mode of operation, the operating window of the injection process fell below the free water formation line (the lower shaded rectangle in Figure 11), and free water was dropped out of the system. Hence, the project was stopped due to the risk of corrosion, and a dehydration plant was installed to inject dry CO_2_. During different transient operations (such as commissioning, well shutdown and restart, water flushing and restart, etc.), it is possible that operating conditions are less than the main condition governing the system.^35^ Therefore, it is imperative that all of the possible scenarios are considered at the process design stage if dwCO_2_ injection is to be implemented.

In such projects with short-distance offshore pipelines, a combination of engineering methods such as the use of CRAs and corrosion inhibitors could be helpful to avoid the corrosion risks and conduct a safe water-saturated CO_2_ injection with a low chance of salt precipitation in the reservoir. The Sleipner CCS project is also performed under water-saturated conditions with the help of CRAs.^32,33,36^

## Discussion

It is widely accepted that prevention is always better than cure, especially that side effects may also be an inevitable part of any treatment method.^20^ The rationale of the proposed methodology in this work is based on this preventive approach by targeting the underlying physics that triggers salt precipitation rather than working a solution method after damage has occurred. The contribution of this study lies in its comprehensive examination of the mechanisms driving salt precipitation and the integration of this understanding into a practical mitigation strategy. The dynamics between the CO_2_-brine-rock interactions and salt precipitation are coupled with the innovative solution of dissolved-water CO_2_ injection (dwCO_2_ injection). By presaturating CO_2_ with water, the water evaporation, which is the key trigger for salt precipitation, is effectively reduced or eliminated. Unlike conventional methods such as fresh water flushing or low-salinity waterflooding, which may introduce long-term reservoir issues like clay swelling and salt recrystallization, dwCO_2_ injection offers a less invasive alternative solution.^20^ The broader implications of this method lie in its ability to balance reservoir management and operational risks, aligning reservoir management strategies with infrastructure considerations. By demonstrating that even modest water content can mitigate salt precipitation effectively, the study provides a pathway for industries to prioritize reservoir management without compromising infrastructure safety.^29^ Additionally, the method will be able to save costs by reducing maintenance expenses associated with salt precipitation damage. By improving storage site performance, this method offers potential economic benefits, reducing downtime and increasing operational efficiency.

Looking forward, the implications of dwCO_2_ injection extend beyond addressing salt precipitation and redefining how CCS projects can be optimized for operational resilience. This approach not only safeguards reservoir performance but also aligns with broader CCS goals of scalability and sustainability, contributing to the global fight against climate change while minimizing risks. That is directly impacted by integrating disciplines such as chemistry, geochemistry, chemical engineering, well, and reservoir engineering to present a novel, comprehensive, and multidisciplinary approach to tackle a pressing problem in CCS technology.

Ultimately, this work contributes to the field by bridging the gap between theoretical modeling and practical application by promoting field-applicable methods to mitigate salt precipitation effects. The study underscores the importance of meticulous engineering and management strategies for successful implementation by addressing the associated challenges, such as corrosion, particularly in scenarios where free water may form due to unforeseen operational changes or transient conditions, as also shown by the Gorgon CCS project.^98^ The discussion of challenges, such as correct process design for corrosion prevention, ensures that the method’s practicality is evaluated within a real-world operational framework, providing actionable insights for industry adoption.

While the simulations presented here validate the effectiveness of dwCO_2_ injection, they also highlight the need for further experimental and field-scale studies. Future research should focus on refining the insights to tailor dwCO_2_ injection for specific reservoir conditions, thereby enhancing its field-scale implementation and validating its potential, adaptability, feasibility, and applicability across diverse CCS projects. Key questions remain in the second injection method regarding the optimal water content for different reservoir conditions, the correct inhibitor compounds, and process design strategies tailored for dwCO_2_ injection scenario.

## Conclusions

This study investigated the intricate processes of CO_2_ storage in a carbonate aquifer, including water evaporation, salt precipitation, CO_2_ dissolution, capillary backflow, and fluid/rock interactions using numerical simulations by CMG-GEM. After demonstrating the complex interplay between these processes and their collective impact, dissolved-water CO_2_ injection (dwCO_2_ injection) was proposed as a method to prevent salt precipitation.

In a CO_2_ storage process in an aquifer, because of liquid/gas equilibrium of the components, the outflow composition of an injection cell stays almost the same with a constant CO_2_-saturated brine phase and a water-saturated gas phase. Dissolving some contents of water in the CO_2_ dense phase and ideally saturating it with water before injection to the reservoir prevents more evaporation in the aquifer and, hence, prevents salt precipitation. Using simulations, it was demonstrated that dissolving water in injected CO_2_ can significantly reduce salt precipitation. The results showed that saturating the CO_2_ stream with water at the reservoir pressure and temperature reduces the precipitation nearly to zero, and dissolving 2000 ppm molar fraction of water into the CO_2_ injection phase decreased the salt precipitation to one-third.

Two moods of injection based on CO_2_ saturation with water were proposed in this study: (1) saturation within the well and (2) saturation at the surface. In the first method, CO_2_ can be fully saturated with water. Safe and successful operation of the second method requires a number of engineering considerations. If the water content stays within the CO_2_ phase, then the risk of corrosion can be controlled. The minimum tolerable water content in the CO_2_ phase stream is highly operational-dependent. In the normal operating conditions, an acceptable amount of water can be dissolved into the CO_2_ stream without concerns regarding water dropout or corrosion and hydrate formation. The presence of some special impurities can reduce the tolerable water content in the CO_2_ stream to very small values. If the process, well, and reservoir engineers conduct an integrated assessment of the possible operating range of their process—from compressor to wellhead and wellhead to sand face—to design an operational condition where the dissolved humidity remains within the CO_2_ dense phase without dropping out, using this method, they can prevent salt precipitation appropriately without needs for further maintenance or any concerns regarding corrosion. Therefore, the knowledge and control of the process are key to this method. In the cases where the window of tolerable water content is very small, a combination of CRAs and corrosion inhibitors can help to make this method practicable. A correct analysis weighs the costs of correct process design and corrosion-prevention measures against the lifelong benefits of reservoir damage prevention, costs of water flush, and disruption to production or injection.

In conclusion, understanding the mutual effects of CO_2_ trapping mechanisms, water evaporation, and salt precipitation is essential for optimizing CO_2_ storage in carbonate aquifers. This study provides valuable insights into these interactions and offers a technical solution to prevent salt precipitation in CO_2_ storage processes in saline aquifers in addition to discussing the related challenges and how to tackle them. Further research and field validations are recommended to assess these findings and implement effective CO_2_ storage strategies.

## Data Availability

All the data for this article are available in the main text.

## Supporting Information Available

Theoretical background, CO_2_ subsurface interactions in an aquifer during a storage process, challenges and feasibility of humid CO_2_ injection (either wsCO_2_ injection or dwCO_2_ injection), dissolution of CO_2_ in brine and rock dissolution/precipitation, simulation of CO_2_ injection in a Middle Eastern carbonate rock, simulation of CO_2_ injection in the SACROC Unit in the Permian basin of western Texas, evolution of pH and dissolved calcite during a CO_2_ storage experiment in two different salinities (0 and 6 mol NaCl), water solubility in CO_2_ at different pressures and temperatures, hydrate dissociation curves for pure CO_2_ and CO_2_-rich fluid in the presence of free water, hydrate dissociation curves in the presence and absence of free water, i.e., dissolved water content, hydrate dissociation curves of three aquifer salinities and the operating conditions of an offshore CO_2_ injection process in the summer and winter time, and water/oil and liquid/gas relative permeability curves and capillary pressure (Pc) curve (PDF)
