# Supplementary material for: Preventing Salt Precipitation in CO2 Storage Processes in Saline Aquifers: Dissolved-Water CO2 Injection Method
Source: Energy Fuels. 2025 Feb 18;39(8):3926–41. doi: 10.1021/acs.energyfuels.4c05249 (PMC11874032; doi:10.1021/acs.energyfuels.4c05249)
Supplement: Supplementary file 1 — ef4c05249_si_001.pdf [file ef4c05249_si_001.pdf]

Supporting Information

Preventing Salt Precipitation in CO_2 Storage Processes in Saline Aquifers: Dissolved-Water CO_2 Injection Method

Ali Papi[*a], Amir Jahanbakhsh*[a, b] *and M. Mercedes Maroto-Valer*[a, b]

[a] Research Centre for Carbon Solutions (RCCS), School of Engineering and Physical Sciences, Heriot-Watt University, UK, EH14 4AS
[b] Industrial Decarbonisation Research and Innovation Centre (IDRIC), Heriot-Watt University, Edinburgh, UK, EH14 4AS

CORRESPONDING AUTHOR

*** Corresponding Author**

Tel.: +44(0)1314518028, *E-mail address:* ali.papi@hw.ac.uk

Figures S1-S9

THEORETICAL BACKGROUND

CO_2 Subsurface Interactions in an Aquifer During a Storage Process. After CO_2 is injected into the aquifer, it dissolves in brine and acidifies it. This nurtures the conditions for rock dissolution and precipitation reactions (**Figure S1**).[1, 2] Sandstone rocks have shown weak potentials for chemical reactions despite presence of some reactive inter-granular materials, while the potentials for carbonate rocks can be strong.[3-6] Calcite and dolomite as the most dominant minerals in carbonate rocks have 3 to 4 orders of magnitude higher reaction rates than most minerals of sandstone rocks.[7]

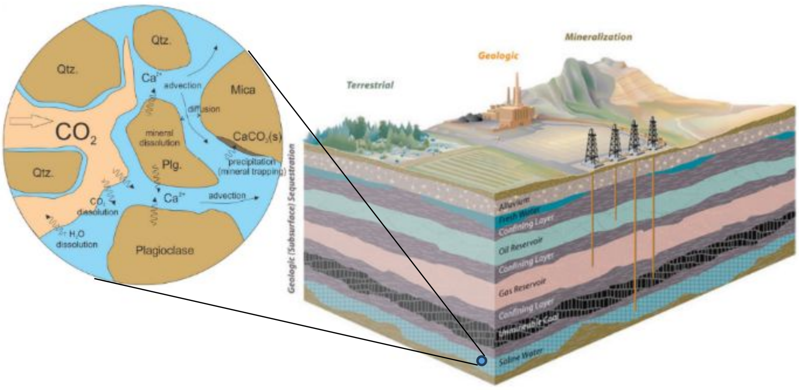

Figure S1. Dissolution of CO_2 in brine and rock dissolution / precipitation. Reproduced or adapted with permission from [1, 2]. Copyright 2013 Mineralogical Society of America and Copyright 2015 Springer.

Having said that, studies have shown that the amount of dissolved or precipitated rock in carbonates can be limited, in order of 0.1 to 10 mol per cubic meter of reservoir rock.[8-10] This is as low as 4 to 6 decimals of rock volume dissolution or precipitation which means that the extent of porosity change due to geochemical reactions can be negligible.[11] **Figure S2** shows the simulation results of CO_2 injection into a Middle Eastern carbonate rock with 95% calcite and 5% dolomite in a brine aquifer with different ions (Na, Cl, K, Ca, Mg, Sr, Fe, C, and S).[5] CO_2 is injected for 30 years and the figure shows the geochemical interactions after 1,000 years. As can be seen, less than 5 mol

calcite per reservoir cubic meter (**Figure S2(a)**) and less than 1 mole dolomite per reservoir cubic meter (**Figure S2(b)**) are dissolved within this timeframe. The maximum porosity change due to the dissolution of these two minerals after 1,000 years is $+6\times10^{-4}$ (**Figure S2(c)**), equal to only 0.06% porosity increase, which is negligible.

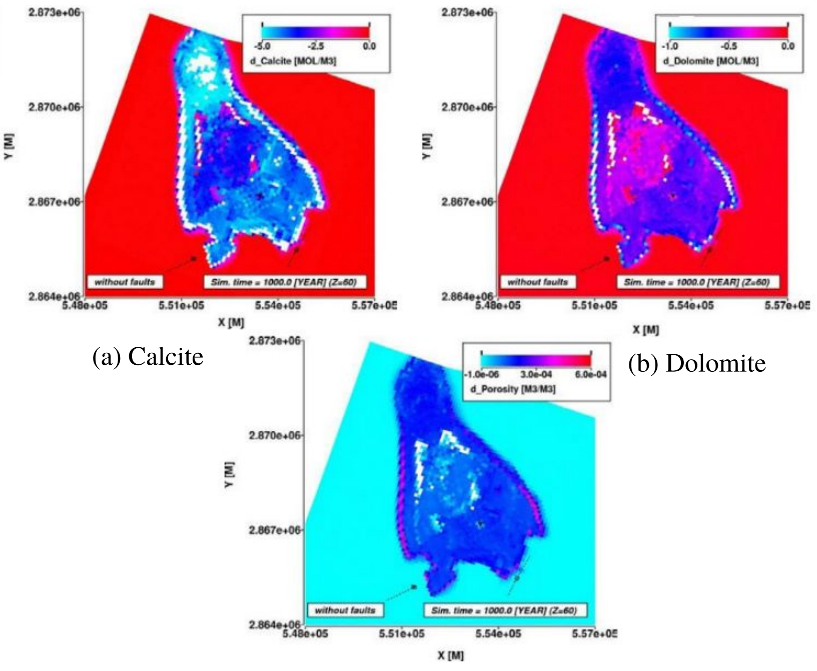

(a) Calcite (b) Dolomite

(c) Porosity

Figure S2. Simulation of CO_2 injection in a Middle Eastern carbonate rock; the changes in (a) calcite, (b) dolomite and (c) porosity after 1,000 years. Reproduced or adapted with permission from [5]. Copyright 2017 Elsevier.

Figure S3 shows the simulation results of the SACROC Unit in the Permian basin of western Texas where CO_2 is injected for 30 years.[11] The aquifer is composed of 62% calcite, 10% dolomite, and other minerals such as ankerite and anhydrite with different dissolved ions in the brine (Na, Cl, K, Ca, Mg, Al, Fe, etc.). The amount of dissolved calcite and precipitated dolomite after 200 years of simulation are shown in **Figures S3(a)** and **S3(b)**, respectively. On average, around 0.2 Kg/m^3 calcite is dissolved and 0.1 Kg/m^3 dolomite is precipitated within this timeframe. This is

respectively equivalent to a rock volume change of $+7.4\times10^{-5}$ (with calcite density equal to 2710 Kg/m^3) and -3.5×10^{-5} (with dolomite density equal to 2865 Kg/m^3) per reservoir volume which means that the average total porosity change due to calcite and dolomite geochemical reactions after 200 years is around 0.004%, a negligible amount.

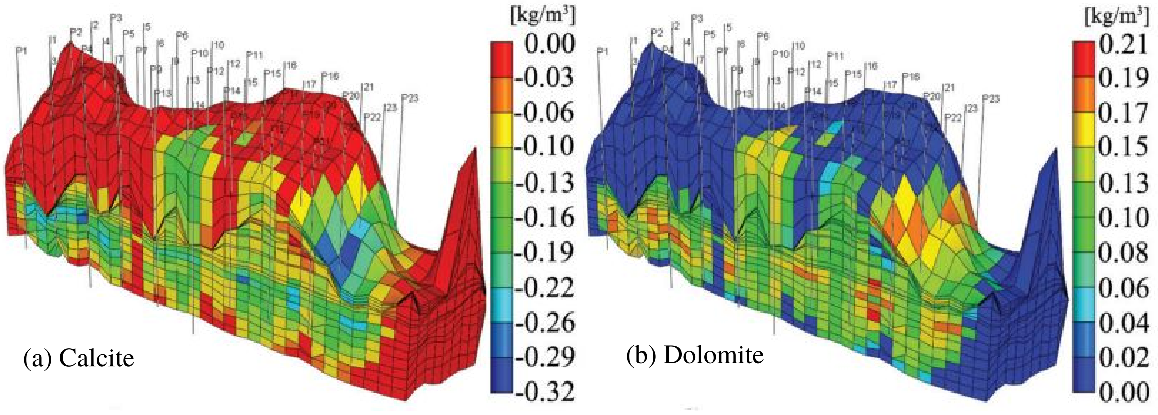

Figure S3. Simulation of CO_2 injection in the SACROC Unit in the Permian basin of western Texas; the changes in (a) calcite and (b) dolomite after 200 years. Reproduced from ref [11]. Available under a CC-BY-SA license. Copyright 2010 Weon Shik Han et al.

These results show that the rock dissolution or precipitation only happens up to a limited extent, which can be of up to 0.01% rock volume change on average. The reason for this behaviour has been demonstrated in an experimental study (**Figure S4**). In this work, CO_2 is injected into a 1 m^3 bulk of pure carbonate rock (calcite) with 0.2 porosity and 50% brine saturation at 80 ˚C and 200 bar at two different salinities. Salinity can increase the CO_2 dissolution and hence, increase the rock dissolution rate by acidifying the brine. The resulting dissolved carbonate rock in this 0.1 m^3 water at its saturation point (2 g/l) is 0.00007 m^3, meaning that the new porosity will only be 20.007% (20.01% for the case of brine).[12]

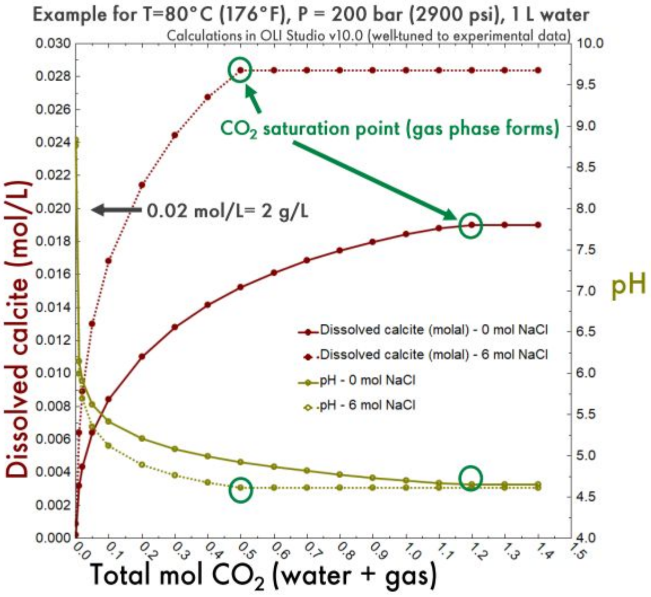

Figure S4. The evolution of pH and dissolved calcite during a CO_2 storage experiment in two different salinities (0 mol and 6 mol NaCl); as the CO_2 is dissolved, pH is reduced and calcite is dissolved in brine, but these trends continue until an equilibrium is reached. Reproduced or adapted with permission from [12].

This is because when CO_2 is dissolved in water and reduces the pH of the system, it only reacts with the formation rock until an equilibrium is reached. This 'spent acid' stays in contact with rock without further reactions while it is fully saturated with the rock ions. As can be seen from **Figure S4**, after some point, no more calcite is dissolved into the brine and the curves (pH curve and dissolved calcite curve) reach a constant line. This should be noted that this phenomenon in supercritical CO_2 injection in aquifers is totally different from the phenomena in water-based CO_2 injection methods such as carbonated-brine CO_2 injection where a fresh acid (brine) is constantly being injected into the subsurface and can dissolve a massive amount of rock.[6, 13, 14] In total, even though mineral trapping constitutes a small portion of the trapping mechanisms, it still plays an important role in CO_2 storage. Especially as the mineral rock is precipitated, it can improve the cap-rock seal's integrity by cementing this shale rock from the surrounding environment.[15]

Due to the high salinity of saline aquifers, compared to other geo-storage methods, these formations pose other challenges to the CO_2 storage process, such as the intensive effect of salt precipitation.[16-19] Saline aquifers contain considerable amounts of Na^+ and Cl^- concentrations that can precipitate when their concentrations are altered due to water evaporation. Evaporation can decrease the water content, and hence, increase the molality of these ions which would lead to salt precipitation if their saturation limit is exceeded. The salting out processes during CO_2 storage in saline aquifers have been looked into extensively.[20-22] Some researchers have conducted geochemical laboratory experiments and, in addition to salt precipitation, have observed different elements in the brine solution due to the reaction of carbonated brine with rock minerals, e.g. with the carbonate minerals that exist in a sandstone.[23-27] Having said that, the CO_2 / rock interaction behaviour in the presence of water evaporation and salt precipitation is not clear. It is believed that the occurrence time scale of these phenomena is different, with salt precipitation happening very fast and ceasing by the stoppage of CO_2 injection after a couple of years, while rock dissolution and precipitation reactions happening very slowly on the scale of years to thousands of years or more after the injection.[28, 29] But these assumptions and interactions need to be investigated and scrutinized further.[30]

Challenges and Feasibility of Humid CO_2 Injection (Either wsCO_2 Injection or dwCO_2 Injection). There are a number of considerations in proposing the idea of humid CO_2 injection (dwCO_2 injection or wsCO_2 injection), one of which is the implications for compression. Changing the CO_2 composition from dry CO_2 can alter the thermodynamics of the injection process by shifting the phase diagram rightward, thereby affecting properties such as viscosity and density.[31, 32] The increase of these properties directly affects the compression as the pressure levels must be carefully

maintained to ensure that CO_2 remains in the supercritical state during compression. Therefore, this can affect the process design, such as designing the correct compressor specifications (as in compressor capacity) as well as pipeline and operation design, so the desired compression be achieved. Apart from compression, an excess amount of water content in the CO_2 dense phase could lead to two-phase flow, condensation or water dropout which are not satisfactory flow assurance circumstances.[33, 34] The presence of free water in the CO_2 stream can cause corrosion and hydrates formation which are among the most important challenges in the context of humid CO_2 injection. Therefore, avoiding the free water formation region is of utmost importance.[35] In the following paragraphs, a detailed analysis will explore strategies to address these challenges.

Corrosion: Numerous experimental studies have been conducted in the literature to investigate the corrosion extent of humid CO_2 in different carbon steels in the presence of different impurities, including CO_2-H_2O-O_2,[36-40] CO_2-H_2O-SO_2-O_2,[41-45] CO_2-H_2O-O_2-NO/NO_2,[38, 46] CO_2-H_2O-H_2S,[38, 47] and complex mixtures such as CO_2-H_2O-NO_2-SO_2-O_2.[46, 48-50] The formation of different acids such as carbonic acid, nitric acid, sulphuric acid and sulphurous acid is known to be the primary cause of corrosion. The use of Corrosion Resistive Alloys (CRA's) such as stainless steel or low Cr steel have shown to be efficient in preventing corrosion in all the impurities mentioned above except in the case of complex mixtures where more attention is required.[47, 51, 52] The coexistence of SO_x and NO_x in the system causes the formation of strong acids that even CRA's cannot resist.[53] The cross-chemical reactions between these impurities cause the formation of nitric acid, sulphuric acid and sulphurous acid that trigger the corrosion at very low impurities concentrations.[37, 49, 50, 53] However, in the absence of these impurities, CRA's have shown to work well for corrosion prevention, but in this case, economic considerations factor in.[46, 53, 54] Transporting gigaton scales of CO_2 for storage

requires multiple high diameter pipelines that can extend tens of kilometres from their pumping and compression point to the injection point through onshore or offshore connections. Due to this reason, using Corrosion Resistive Alloys (CRA's) is not economically viable for long-distance pipelines. So, less expensive materials such as carbon steel are needed.[46] Corrosion inhibitors can play a role in these circumstances. Amines are an example but there is no universally accepted amine compound that can inhibit corrosion in all operational conditions. Corrosion inhibitors have to be tailored to the specific process and their effectiveness is restricted to some desired properties such as boiling point, vapour-liquid equilibrium, their salt formation potential, etc. Corrosion inhibitors have been extensively studied in oil and gas industry but there are huge differences between oil and gas industry and CO_2 transport because the operational conditions, dominant phase and contaminants are different. For example, there is no guarantee that the same amine effective in preventing corrosion in the oil and gas industry would also work to inhibit corrosion during the pipeline transportation of CO_2 in a supercritical dense phase with low water content.[53] More studies are required to be conducted in this field to create tailored inhibitors for CCS applications.[55, 56] Due to these limitations, other engineering methods in the process design should be considered, as discussed in the following.

It is important to note that corrosion in CO_2 transportation infrastructures becomes an issue only when free water is present in the CO_2 stream.[53] It is in such circumstances that CO_2 (or other impurities) would dissolve in the free water and create carbonic acid (or other acids) which is corrosive.[8, 35] In another word, as long as the dissolved water content stays within the CO_2 phase, corrosion risks are minimal.[53, 57] It is noteworthy to mention that CO_2 has a certain capacity to dissolve water in its stream (water solubility in CO_2) which is a function of pressure and

temperature.[35, 46, 58] When this solubility limit is exceeded, water drops out of the CO_2 solution and is transported in the form of free water. Thus, there is a tolerable limit for humid CO_2 without causing water dropout and corrosion risks. Therefore, corrosion prevention in the context of dwCO_2 injection with second injection method is translated into limiting the water content in the CO_2 stream within its solubility limit.[59]

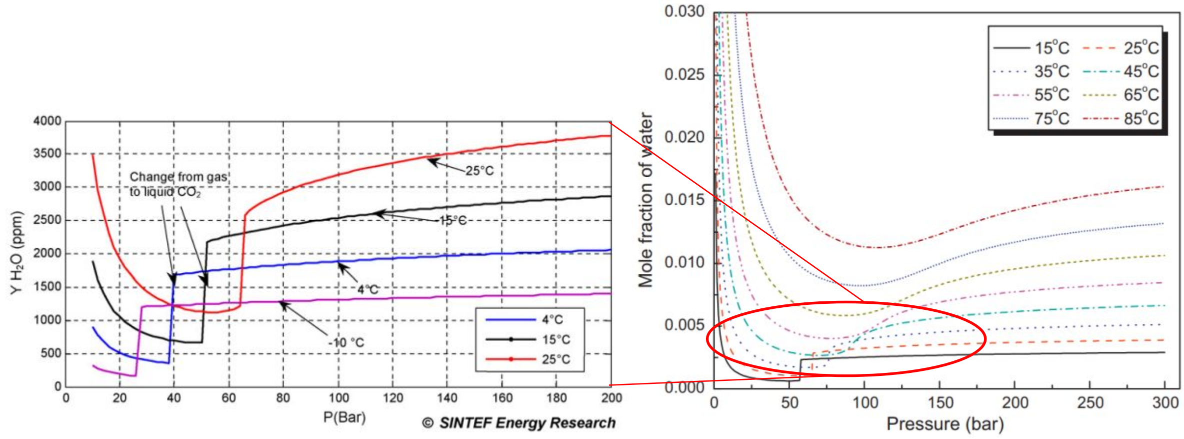

Figure S5. Water solubility in CO_2 at different pressures and temperatures. Reproduced or adapted with permission from [34, 35] Copyright 2008 and 2011 Elsevier.

The plot of water solubility in CO_2 at different temperatures and pressures is shown in **Figure S5** where y_{H2O} is the mole fraction of water that can be dissolved in the CO_2 phase.[18, 34, 35, 58, 59] **Figure S5** (left) is an enlarged image of the **Figure S5** (right) that puts the focus on the temperature range of -10 °C to 25 °C which can be a bottleneck in a CO_2 injection process. The solubility of water in CO_2 monotonically increases with temperature and pressure when CO_2 is in the supercritical state. The injected CO_2 is in supercritical condition in the reservoir temperature and pressure range of 25 ˚C to 200 ˚C and 10-300 bar.[60, 61] Based on **Figure S5** (left), beyond 1300 molar ppm water can be dissolved in the CO_2 dense phase in the supercritical condition above 71.3 bar and the temperature

range of -10 °C to 25 °C. This means that beyond 1300 ppm water can be tolerated as dissolved in CO_2 in these conditions without causing water dropout and corrosion. However, this is the best-case scenario; worst-case scenarios which are more conservative and lower must be considered. Before CO_2 reaches the reservoir, it can undergo a series of thermodynamic changes by passing through different facilities and environments. These changes can alter the physics state of the CO_2 dense phase from the supercritical condition, reducing its water solubility limit. Therefore, it is crucial to factor in all the potential operational conditions of the storage process, from the surface to subsurface. If the process is designed solely for supercritical conditions and overlooks parts of the operation that may deviate from these conditions, water could drop out of the system and can lead to corrosion. So, all the possible scenarios should be accounted for.

If the pipelines operate at the seabed temperature of 4 °C, the pessimistic value of water solubility limit can be as low as 350 molar ppm at around 40 bar, a non-supercritical condition. If the pipelines are buried in soil, the operating temperatures are between 5-10°C with a worst-case scenario water solubility limit of less than 650 molar ppm. Operating at the ambient temperature can give different minimum values depending on winter- or summer-time operations. As an example, operating at 25°C allows water contents present in the CO_2 phase of up to 1200 molar ppm without any free water dropout or corrosion concerns. This value can be beyond 2500 molar ppm water if the system operates at supercritical condition. Due to the risk of Joule-Thomson effect, operating conditions at or below -10°C are possible near the sandface which can have a pessimistic water solubility value of less than 200 ppm.[57] On the other hand, the presence of SO_x and NO_x impurities in the CO_2 phase stream can lead to risk of severe corrosion in dissolved water contents much below the pessimistic values mentioned above. These impurities react with water and produce sulphuric acid, sulphurous

acid and nitric acid.[37, 49, 50, 53] Due to these reasons, there is no universally fixed value for the minimum tolerable water content in the CO_2 phase stream in a storage process.[35, 46, 53] As was shown, this value is highly operational-dependent and is different from case to case, e.g. operating onshore, offshore, under extreme or normal weather conditions, under presence of certain impurities, etc. A safe scenario is that water would be completely removed from the CO_2 stream and for this reason, water contents of less than 50 ppm are advised in some references.[53, 57] Various water contents from 50 ppm to 650 ppm have been used in different CO_2 transportation projects across the world.[53, 62-64] The Sleipner CO_2 storage process has been operated under water-saturated conditions with the benefit of Corrosion Resistive Alloys (CRA's).[35, 53, 62]

Hydrates: Compared to corrosion, the risk of hydrate formation during CO_2 storage is not significant but it is a possibility.[57] The conditions for formation of hydrates are more moderate and there are some measures in place to inhibit this phenomenon, if occurred. Hydrates typically form under low temperatures and high pressures. The hydrate dissociation curves for pure CO_2 and CO_2-rich fluid (96% > CO_2) in the presence of free water is shown in **Figure S6**. Temperatures less than 10°C are required for hydrates to be formed in such circumstances. In the onshore projects with ambient temperatures of above 10 °C (or even less), chances of hydrate formations are null. Therefore, given the operational conditions of CO_2 injection in deep saline aquifers, hydrate formation is not considered a frequent or severe issue.[31, 57]

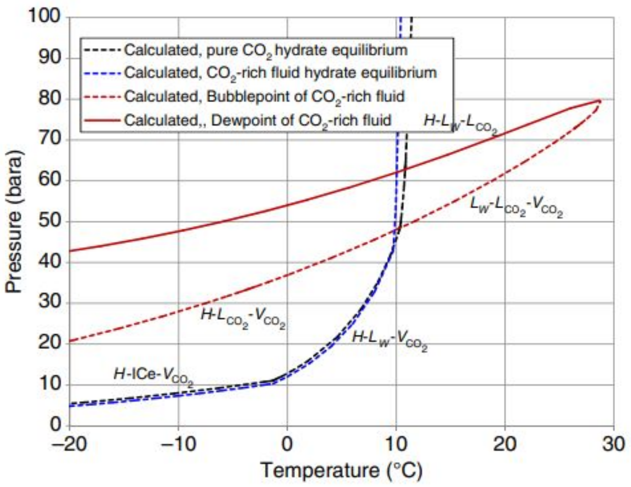

Figure S6. Hydrate dissociation curves for pure CO_2 and CO_2-rich fluid in the presence of free water. Reproduced from ref [57]. Available under a CC-BY-SA license. Copyright 2015 Yang et al.

Unlike corrosion, the formation of hydrates can occur not only in the conditions of free water, but also in the absence of it, meaning that hydrates can form even when water is dissolved in the CO_2 dense phase.[31] As can be seen in **Figure S7**, in the case of dissolved water (no free water), the hydrate formation curve shifts leftwards to lower temperatures. This shift makes the conditions less favourable and more challenging for hydrate formation, that can be helpful to facilitate its control. In an offshore injection facility with a seabed injection temperature of 4 °C, the risk of hydrate formation in the absence of free water in the range of water contents shown in **Figure S7** does not exist, as this operational condition lies outside of the hydrate dissociation curve. In the following, an example from the literature with more detailed analysis will be provided.

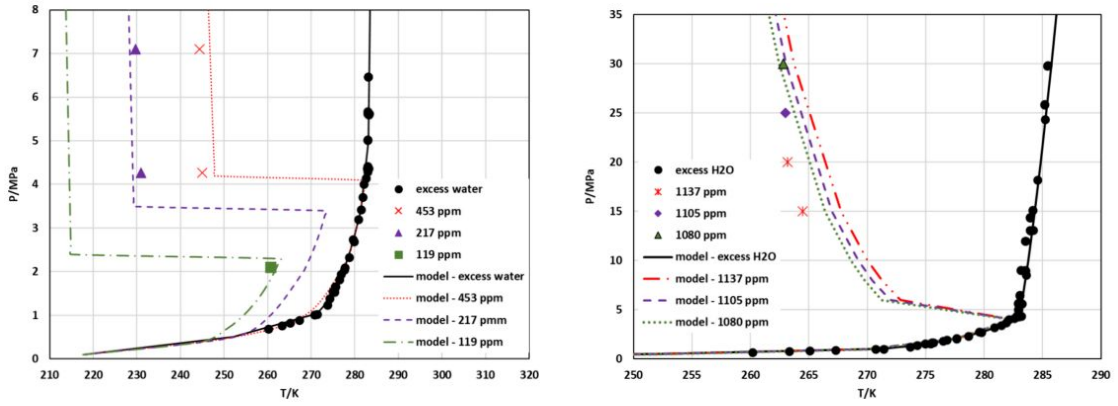

Figure S7. Hydrate dissociation curves in the presence and absence of free water, i.e. dissolved water content. Reproduced from [31]. Copyright 2024 American Chemical Society.

These analyses exclude the possibility of Joule-Thomson effect and transient well operations.[57] Joule-Thomson effects can reduce the CO_2 stream temperatures to values that lie within the risk of hydrate formation. In such cases, prevention measures such as hydrate inhibitors are needed. Joule-Thomson cooling happens when the CO_2 stream undergoes a huge pressure drop and as a result, it expands and cools down, such as when it enters the reservoir from the well.[65, 66] Therefore, the pressure change across the sandface needs to be controlled in order to avoid this effect. Depleted gas reservoirs are more prone to this phenomenon as opposed to deep saline aquifers.[67] The reservoir pressure in depleted gas reservoirs has been exhausted and the pressure in these reservoirs is low. Therefore, there are limitations on the magnitude of the injection pressure. If the injection pressure is much more than the reservoir pressure, CO_2 can see Joule-Thomson cooling. In such cases, hydrate inhibitors are required. Monoethylene glycol (MEG) and methanol are preferred thermodynamic options. However, because of the relatively high freezing temperature of MEG, it is not recommended for use in depleted gas reservoirs where very low temperatures are expected.

Methanol, on the other hand, is not advised for deep saline aquifers because of its adverse effects on the salting out process in these brine environments.[57]

Example – hydrate formation: **Figure S8** shows the hydrate dissociation curves and the operating conditions of an offshore CO_2 injection process into a deep saline aquifer in the literature.[57] The red lines and blue lines show the operating conditions in the summer and winter, respectively (from pump station to the platform, from platform to wellhead choke and from choke to bottomhole). The dashed lines show the free water hydrate dissociation curves for 3 salinities: pure water (zero salinity), 60 g/l saline aquifer and 180 g/l saline aquifer. It is worth mentioning that salt acts as a corrosion inhibitor.[31] The hydrate dissociation curve shifts leftward to lower temperatures when there is salt in the free water. As can be seen from the figure, the summer-time operational conditions entirely lie outside of the hydrate formation limit therefore during these times, there is no chance of hydrate formation. On the other hand, during the winter-time operations, seabed temperatures of down to 4°C are expected near the wellhead choke. In the zero-saline aquifer, this could be a risk of hydrate formation but because there is a salinity of 180 g/l in this aquifer, the hydrate dissociation curve is shifted to lower temperatures and under these conditions, no hydrate formation is occurred in the winter-time. On the other hand, when CO_2 is injected into the well from the wellhead, its temperature and pressure increase as it reaches to the sandface. This counts as a favourable condition for the prevention of hydrate formation as in this case, the salinities of down to 60 g/l can tolerate the injection process without hydrate formation. So, no hydrate is formed anywhere in this system from the pump station to bottomhole, neither in the trunkline and infield flowlines nor in the injection well, in the summer or winter.

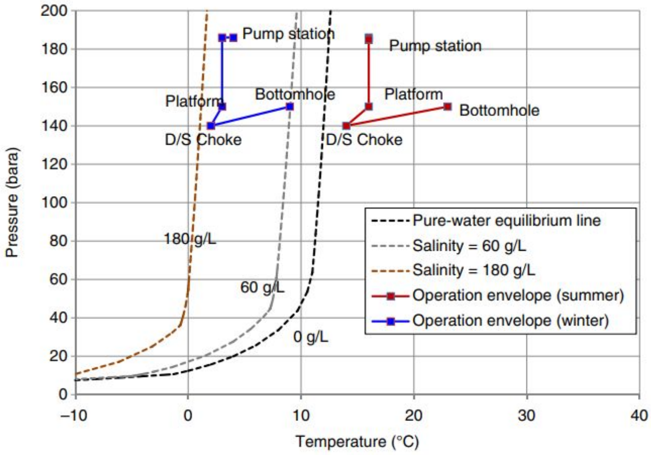

Figure S8. Hydrate dissociation curves of three aquifer salinities and the operating conditions of an offshore CO_2 injection process in the summer and winter time. Reproduced from ref [57]. Available under a CC-BY-SA license. Copyright 2015 Yang et al.

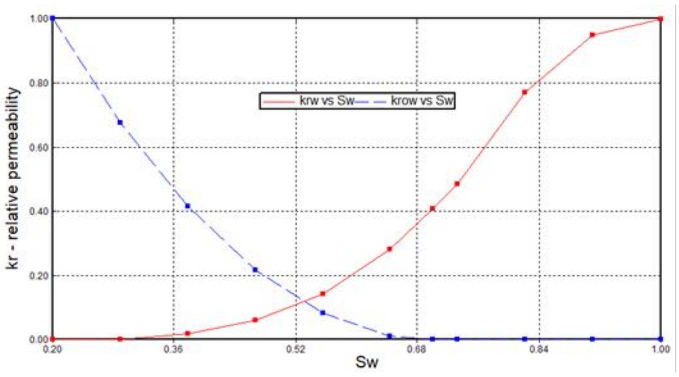

(a)

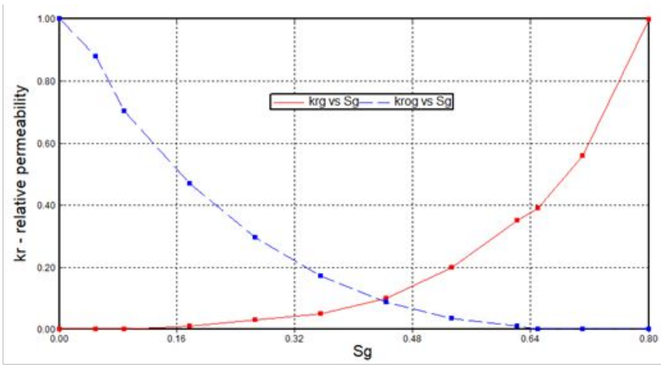

(b)

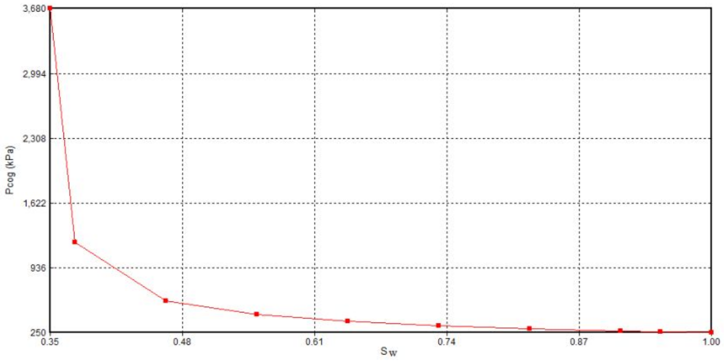

(c)

Figure S9. (a) Water/oil and (b) liquid/gas relative permeability curves and (c) capillary pressure (Pc) curve.

References

(1) Steefel, C. I.; Molins, S.; Trebotich, D. Pore scale processes associated with subsurface CO2 injection and sequestration. *Reviews in Mineralogy and Geochemistry* **2013**, *77* (1), 259-303.
(2) Farmer, G. T. *Modern climate change science: an overview of today's climate change science*; Springer, 2015.
(3) Romanov, V.; Soong, Y.; Carney, C.; Rush, G. E.; Nielsen, B.; O'Connor, W. Mineralization of carbon dioxide: a literature review. *ChemBioEng Reviews* **2015**, *2* (4), 231-256.
(4) Izadpanahi, A.; Blunt, M. J.; Kumar, N.; Ali, M.; Tassinari, C. C. G.; Sampaio, M. A. A review of carbon storage in saline aquifers: Mechanisms, prerequisites, and key considerations. *Fuel* **2024**, *369*, 131744.
(5) Tambach, T. J.; Lonnee, J.; Snippe, J. R. Forecast of reactive CO2 injection into a carbonate formation, Middle East. *Energy Procedia* **2017**, *114*, 2847-2855.
(6) An, S.; Erfani, H.; Hellevang, H.; Niasar, V. Lattice-Boltzmann simulation of dissolution of carbonate rock during CO2-saturated brine injection. *Chemical Engineering Journal* **2021**, *408*, 127235.
(7) Palandri, J. L.; Kharaka, Y. K. *A compilation of rate parameters of water-mineral interaction kinetics for application to geochemical modeling*; US Geological Survey, 2004.
(8) Erfani, H.; Babaei, M.; Niasar, V. Dynamics of CO2 density-driven flow in carbonate aquifers: Effects of dispersion and geochemistry. *Water Resources Research* **2021**, *57* (4), e2020WR027829.
(9) Islam, A.; Sun, A. Y.; Yang, C. Reactive transport modeling of the enhancement of density-driven CO2 convective mixing in carbonate aquifers and its potential implication on geological carbon sequestration. *Scientific reports* **2016**, *6* (1), 24768.
(10) Fu, X.; Cueto-Felgueroso, L.; Bolster, D.; Juanes, R. Rock dissolution patterns and geochemical shutdown of–brine–carbonate reactions during convective mixing in porous media. *Journal of Fluid Mechanics* **2015**, *764*, 296-315.
(11) Han, W. S.; McPherson, B. J.; Lichtner, P. C.; Wang, F. P. Evaluation of trapping mechanisms in geologic CO 2 sequestration: Case study of SACROC northern platform, a 35-year CO 2 injection site. *American Journal of Science* **2010**, *310* (4), 282-324.
(12) Snippe, J.; Tucker, O.; Ardill, A. The effective reactivity of carbonate rock with injected $CO\text{-}_2$ – from Core Scale to Field Scale. In Mini-Symposium on Subsurface Porous Media, Manchester; 2024.
(13) Menke, H. P.; Bijeljic, B.; Andrew, M. G.; Blunt, M. J. Dynamic three-dimensional pore-scale imaging of reaction in a carbonate at reservoir conditions. *Environmental science & technology* **2015**, *49* (7), 4407-4414.
(14) Chen, Y.; Clennell, B.; Zhang, J.; Tang, M.; Ahmed, S. Reactive transport modelling of in-situ CO2 mineralization in basalt formations. *Capillarity* **2024**, *13* (2), 37-46.
(15) Johnson, J. W.; Nitao, J. J. Enhanced caprock integrity and selfsealing of the immiscible plume through mineral trapping during prograde and retrograde CO2 sequestration in saline aquifers. *AAPG Bull* **2002**, *86*, 161-162.
(16) Miri, R.; van Noort, R.; Aagaard, P.; Hellevang, H. New insights on the physics of salt precipitation during injection of CO2 into saline aquifers. *International Journal of Greenhouse Gas Control* **2015**, *43*, 10-21.
(17) Wen, G.; Li, Z.; Long, Q.; Azizzadenesheli, K.; Anandkumar, A.; Benson, S. M. Real-time high-resolution CO 2 geological storage prediction using nested Fourier neural operators. *Energy & Environmental Science* **2023**, *16* (4), 1732-1741.
(18) Nooraiepour, M.; Fazeli, H.; Miri, R.; Hellevang, H. Effect of CO2 phase states and flow rate on salt precipitation in shale caprocks—a microfluidic study. *Environmental science & technology* **2018**, *52* (10), 6050-6060.

(19) Wang, Z.; Chen, S.; Yuan, H.; Hong, Z.; Xu, R.; Jiang, P.; He, D. Experimental Investigation on Salt Precipitation Behavior during Carbon Geological Sequestration: Considering the Influence of Formation Boundary Solutions. *Energy & Fuels* **2023**, *38* (1), 514-525.
(20) Akindipe, D.; Saraji, S.; Piri, M. Salt precipitation during geological sequestration of supercritical CO2 in saline aquifers: A pore-scale experimental investigation. *Advances in Water Resources* **2021**, *155*, 104011.
(21) He, D.; Wang, Z.; Yuan, H.; Zhang, M.; Hong, Z.; Xu, R.; Jiang, P.; Chen, S. Experimental investigation of salt precipitation behavior and its impact on injectivity under variable injection operating conditions. *Gas Science and Engineering* **2024**, *121*, 205198.
(22) Lopez, O.; Youssef, S.; Estublier, A.; Alvestad, J.; Strandli, C. W. Permeability alteration by salt precipitation: numerical and experimental investigation using X-Ray Radiography. In *E3S Web of Conferences*, 2020; EDP Sciences: Vol. 146, pp 03001, 03010.01051/e03003sconf/202014603001.
(23) Bacci, G.; Korre, A.; Durucan, S. Experimental investigation into salt precipitation during CO2 injection in saline aquifers. *Energy Procedia* **2011**, *4*, 4450-4456.
(24) Xu, T.; Feng, G.; Shi, Y. On fluid–rock chemical interaction in CO2-based geothermal systems. *Journal of Geochemical Exploration* **2014**, *144*, 179-193.
(25) Ueda, A.; Kato, K.; Ohsumi, T.; Yajima, T.; Ito, H.; Kaieda, H.; Metcalfe, R.; Takase, H. Experimental studies of CO2-rock interaction at elevated temperatures under hydrothermal conditions. *Geochemical Journal* **2005**, *39* (5), 417-425.
(26) Cui, G.; Zhang, L.; Tan, C.; Ren, S.; Zhuang, Y.; Enechukwu, C. Injection of supercritical CO2 for geothermal exploitation from sandstone and carbonate reservoirs: CO2–water–rock interactions and their effects. *Journal of CO2 Utilization* **2017**, *20*, 113-128.
(27) Cui, G.; Hu, Z.; Ning, F.; Jiang, S.; Wang, R. A review of salt precipitation during CO2 injection into saline aquifers and its potential impact on carbon sequestration projects in China. *Fuel* **2023**, *334*, 126615.
(28) Grimm Lima, M.; Schädle, P.; Green, C. P.; Vogler, D.; Saar, M. O.; Kong, X. Z. Permeability Impairment and Salt Precipitation Patterns During CO 2 Injection Into Single Natural Brine-Filled Fractures. *Water Resources Research* **2020**, *56* (8), e2020WR027213.
(29) Perkins, E.; Czernichowski-Lauriol, I.; Azaroual, M.; Durst, P. Long term predictions of CO2 storage by mineral and solubility trapping in the Weyburn Midale Reservoir. In *Greenhouse Gas Control Technologies 7*, Elsevier, 2005; pp 2093-2096.
(30) Ott, H.; Roels, S.; De Kloe, K. Salt precipitation due to supercritical gas injection: I. Capillary-driven flow in unimodal sandstone. *International Journal of Greenhouse Gas Control* **2015**, *43*, 247-255.
(31) Queimada, A. J.; Zhang, X.; Pedrosa, N.; Salimi, B. Effect of Salts, Impurities, and Low Water Contents in the Formation of Gas Hydrates in CO2-Rich Streams. *Journal of Chemical & Engineering Data* **2024**, 3284-3295.
(32) Luna-Ortiz, E.; Yao, C.; Barnes, J.; Winter, M.; Healey, M. Development of A CO2 Specification for Industrial CCS Transport Networks: Methodology, Limitations and Opportunities. In *Offshore Technology Conference*, 2022; OTC: p D021S025R004.
(33) Yazdanpanah, M.; Galliot, L.; Perrin, S.; Moulie, G.; Raurich, S. Review of CO2 Specifications in Novel Industrial CCS Transportation and Storage Hubs. In *Abu Dhabi International Petroleum Exhibition and Conference*, 2024; SPE: p D021S050R004.
(34) Choi, Y.-S.; Nešić, S. Determining the corrosive potential of CO2 transport pipeline in high pCO2–water environments. *International Journal of Greenhouse Gas Control* **2011**, *5* (4), 788-797.
(35) De Visser, E.; Hendriks, C.; Barrio, M.; Mølnvik, M. J.; de Koeijer, G.; Liljemark, S.; Le Gallo, Y. Dynamis CO2 quality recommendations. *International journal of greenhouse gas control* **2008**, *2* (4), 478-484.
(36) Sim, S.; Bocher, F.; Cole, I. S.; Chen, X.-B.; Birbilis, N. Investigating the effect of water content in supercritical CO2 as relevant to the corrosion of carbon capture and storage pipelines. *Corrosion* **2014**, *70* (2), 185-195.

(37) Choi, Y.-S.; Nesic, S.; Young, D. Effect of impurities on the corrosion behavior of CO2 transmission pipeline steel in supercritical CO2− water environments. *Environmental science & technology* **2010**, *44* (23), 9233-9238.
(38) Brown, J.; Graver, B.; Gulbrandsen, E.; Dugstad, A.; Morland, B. Update of DNV recommended practice RP-J202 with focus on CO2 corrosion with impurities. *Energy Procedia* **2014**, *63*, 2432-2441.
(39) Hua, Y.; Barker, R.; Neville, A. Effect of temperature on the critical water content for general and localised corrosion of X65 carbon steel in the transport of supercritical CO2. *International Journal of Greenhouse Gas Control* **2014**, *31*, 48-60.
(40) Hua, Y.; Barker, R.; Neville, A. The effect of O2 content on the corrosion behaviour of X65 and 5Cr in water-containing supercritical CO2 environments. *Applied Surface Science* **2015**, *356*, 499-511.
(41) Dugstad, A.; Morland, B.; Clausen, S. Corrosion of transport pipelines for CO2–effect of water ingress. *Energy Procedia* **2011**, *4*, 3063-3070.
(42) Hua, Y.; Barker, R.; Neville, A. The influence of SO2 on the tolerable water content to avoid pipeline corrosion during the transportation of supercritical CO2. *International Journal of Greenhouse Gas Control* **2015**, *37*, 412-423.
(43) Choi, Y.-S.; Nešic, S. Effect of water content on the corrosion behavior of carbon steel in supercritical CO2 phase with impurities. In *NACE CORROSION*, 2011; NACE: pp NACE-11377.
(44) Farelas, F.; Choi, Y.; Nešić, S. Corrosion behavior of API 5L X65 carbon steel under supercritical and liquid carbon dioxide phases in the presence of water and sulfur dioxide. *Corrosion* **2013**, *69* (3), 243-250.
(45) Xiang, Y.; Wang, Z.; Yang, X.; Li, Z.; Ni, W. The upper limit of moisture content for supercritical CO2 pipeline transport. *The Journal of Supercritical Fluids* **2012**, *67*, 14-21.
(46) Dugstad, A.; Halseid, M.; Morland, B. Effect of SO2 and NO2 on corrosion and solid formation in dense phase CO2 pipelines. *Energy Procedia* **2013**, *37*, 2877-2887.
(47) Choi, Y.-S.; Hassani, S.; Vu, T. N.; Nešić, S.; Abas, A. Z. B. Effect of H2S on the corrosion behavior of pipeline steels in supercritical and liquid CO2 environments. *Corrosion* **2016**, *72* (8), 999-1009.
(48) Paschke, B.; Kather, A. Corrosion of pipeline and compressor materials due to impurities in separated CO2 from fossil-fuelled power plants. *Energy Procedia* **2012**, *23*, 207-215.
(49) Yevtushenko, O.; Bäßler, R.; Carrillo-Salgado, I. Corrosion stability of piping steels in a circulating supercritical impure CO2 environment. In *NACE CORROSION*, 2013; NACE: pp NACE-2013-2372.
(50) Yevtushenko, O.; Bä ßler, R. Water impact on corrosion resistance of pipeline steels in circulating supercritical CO2 with SO2-and NO2-impurities. In *NACE CORROSION*, 2014; NACE: pp NACE-2014-3838.
(51) Metz, B.; Davidson, O.; De Coninck, H.; Loos, M.; Meyer, L. Carbon dioxide capture and storage. Summary for policymakers. **2005**.
(52) Sim, S.; Cole, I. S.; Choi, Y.-S.; Birbilis, N. A review of the protection strategies against internal corrosion for the safe transport of supercritical CO2 via steel pipelines for CCS purposes. *International Journal of Greenhouse Gas Control* **2014**, *29*, 185-199.
(53) Barker, R.; Hua, Y.; Neville, A. Internal corrosion of carbon steel pipelines for dense-phase CO2 transport in carbon capture and storage (CCS)–a review. *International Materials Reviews* **2017**, *62* (1), 1-31.
(54) Wei, L.; Pang, X.; Gao, K. Corrosion of low alloy steel and stainless steel in supercritical CO2/H2O/H2S systems. *Corrosion Science* **2016**, *111*, 637-648.
(55) Cen, H.; Cao, J.; Chen, Z.; Guo, X. 2-Mercaptobenzothiazole as a corrosion inhibitor for carbon steel in supercritical CO2-H2O condition. *Applied Surface Science* **2019**, *476*, 422-434.
(56) Kai, Y.; XIANG, Y.; Xiaoling, C. Investigation on corro- sion characteristics of pipeline in CO2 ocean storage system. *Corrosion Science and Protection Technology* **2019**, *31* (6), 672-680.
(57) Yang, S. O.; Hamilton, S.; Nixon, R.; De Silva, R. Prevention of hydrate formation in wells injecting CO2 into the saline aquifer. *SPE Production & Operations* **2015**, *30* (01), 52-58.

(58) Austegard, A.; Barrio, M. Project Internal Memo DYNAMIS: Inert components, solubility of water in CO2 and mixtures of CO2 and CO2 hydrates. *Trondheim, Norway* **2006**.
(59) Cole, I. S.; Corrigan, P.; Sim, S.; Birbilis, N. Corrosion of pipelines used for CO2 transport in CCS: Is it a real problem? *International Journal of Greenhouse Gas Control* **2011**, *5* (4), 749-756.
(60) Adams, J.; Bachu, S. Equations of state for basin geofluids: algorithm review and intercomparison for brines. *Geofluids* **2002**, *2* (4), 257-271.
(61) Bachu, S. Screening and ranking of sedimentary basins for sequestration of CO2 in geological media in response to climate change. *Environmental Geology* **2003**, *44* (3), 277-289.
(62) Boot-Handford, M. E.; Abanades, J. C.; Anthony, E. J.; Blunt, M. J.; Brandani, S.; Mac Dowell, N.; Fernández, J. R.; Ferrari, M.-C.; Gross, R.; Hallett, J. P. Carbon capture and storage update. *Energy & Environmental Science* **2014**, *7* (1), 130-189.
(63) Gale, J.; Davison, J. Transmission of CO2—safety and economic considerations. *Energy* **2004**, *29* (9-10), 1319-1328.
(64) Oosterkamp, A.; Ramsen, J. State-of-the-art overview of CO2 pipeline transport with relevance to offshore pipelines. *Polytec Report number: POL-O-2007-138-A* **2008**.
(65) Roebuck, J.; Murrell, T.; Miller, E. The Joule-Thomson effect in carbon dioxide. *Journal of the American Chemical Society* **1942**, *64* (2), 400-411.
(66) Gao, M.; Wang, L.; Chen, X.; Wei, X.; Liang, J.; Li, L. Joule–Thomson effect on a CCS-relevant (CO2+ N2) system. *ACS omega* **2021**, *6* (14), 9857-9867.
(67) Ziabakhsh-Ganji, Z.; Kooi, H. Sensitivity of Joule–Thomson cooling to impure CO2 injection in depleted gas reservoirs. *Applied energy* **2014**, *113*, 434-451.
